# Can breakthroughs in dermal and transdermal macromolecule delivery surmount existing barriers and revolutionize future therapeutics?

**DOI:** 10.1186/s12967-025-06219-6

**Published:** 2025-05-07

**Authors:** Medha Bhat, Abhay Tharmatt, Samarth Bhargava, Tushar Kumeria, Amit Mishra, Anupama Mittal, Deepak Chitkara

**Affiliations:** 1 Department of Pharmacy, Birla Institute of Technology and Science Pilani, Pilani Campus, Vidya Vihar, Pilani, 333 031 Rajasthan India; 2 School of Materials Science and Engineering, University of New South Wales-Sydney, New South Wales, Australia; 3 Department of Bioscience & Bioengineering, Indian Institute of Technology, Jodhpur, Rajasthan India; 4 Present Address: Department of Pharmaceutics, National Institute of Pharmaceutical Education and Research, SAS Nagar, Punjab, 160062 India

**Keywords:** Dermal delivery, Transdermal systems, Macromolecules, Penetration enhancers, Nanotechnology

## Abstract

The delivery of macromolecules through dermal and transdermal routes presents both significant challenges and transformative opportunities in therapeutic applications. This review highlights the most recent advancements and innovative strategies aimed at overcoming the barriers associated with macromolecular delivery. Cutting-edge approaches such as the use of adjuvants (e.g., hyaluronic acid-based and chemical penetration enhancers), bioactive peptides with diverse functionalities, and mechanical force techniques—including iontophoresis, microneedles, and electroporation—are thoroughly explored. While various strategies have been implemented to enhance skin delivery, they often present significant challenges, particularly for macromolecules. Addressing these challenges requires integrating novel technologies and understanding the interplay between biological barriers and delivery mechanisms. Furthermore, the role of nanotechnology, through systems like nanoemulsions, polymeric nanoparticles, and transferosomes, is examined for its ability to protect macromolecules and regulate their release. Notably, this review provides unique perspectives on the interplay between these strategies and their potential to revolutionise future therapeutics. By highlighting key trends and advancements in macromolecule delivery, this review underscores the importance of innovative approaches in overcoming existing barriers and enabling efficient drug administration.

**Graphical Abstract:**

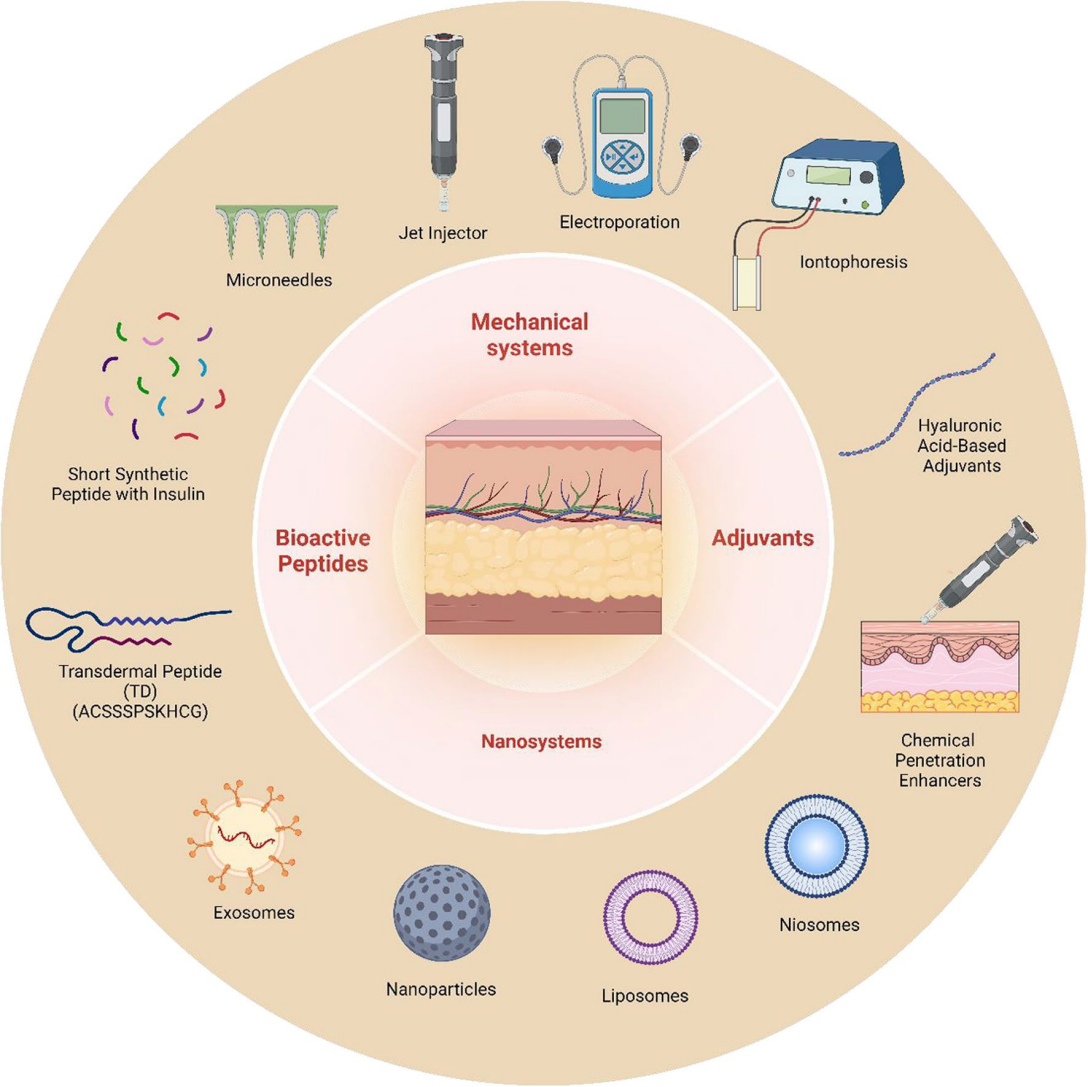

## Introduction

The delivery of macromolecules through the skin has emerged as a promising avenue in drug delivery, involving the administration of peptides, proteins, and nucleic acids, which are particularly valuable due to their high potency and target specificity [1]. However, these macromolecules face significant limitations, including susceptibility to enzymatic degradation and poor membrane permeability due to their large size and hydrophilic nature [2]. The drive behind exploring innovative delivery methods for macromolecules originates from the constraints inherent in conventional therapeutic strategies [3]. For example, the traditional way of delivering macromolecules can result in unintended effects and diminished effectiveness of the treatment. Dermal and transdermal delivery methods can potentially enhance patient adherence and treatment outcomes by circumventing the need for injections and oral administration [4].

In recent years, significant progress has been made in advancing delivery methods specifically designed to enhance the efficacy of macromolecules. The stratum corneum, the outermost layer of the skin, acts as a protective barrier, limiting the penetration of xenobiotics, including macromolecular therapeutics [1]. Its dense and lipophilic nature interferes with the penetration of hydrophilic macromolecules, impeding their transdermal delivery. Moreover, cutaneous disorders such as psoriasis and eczema further complicate drug transport, necessitating innovative delivery strategies [5]. The conventional strategies for topical delivery often involve the utilization of topical formulations, such as creams and ointments, to achieve prolonged contact and localized administration.

Conventional formulations for delivering macromolecules face limitations like insufficient skin penetration and restricted bioavailability. Additionally, they may cause unintended consequences and adverse effects, prompting the development of new techniques to overcome these challenges. Chemical penetration enhancers are used to modify the skin barrier's physicochemical characteristics, facilitating drug permeation [6]. The potential for improving skin penetration exists; however, the application of these substances is impeded by notable limitations, which encompass skin irritation, the formidable barrier presented by the skin through physical modalities and alterations in skin integrity, which consequently restrict their widespread application within clinical settings [7].

Mechanical strategies for macromolecule delivery were employed to modify these limitations into assets facilitating the enhanced penetration of therapeutics. A notable technique entails the utilization of microneedles, which are employed to create microchannels within the skin. This process aids in transporting macromolecules by providing a pathway for their delivery [8]. This particular approach optimizes macromolecular delivery's efficacy and exhibits the potential to alleviate pain and reduce the probability of infection. Another potential approach that may be considered is the utilization of iontophoresis. This mechanical technique harnesses the application of electrical currents to promote the permeation of macromolecules across the skin barrier. Following that, electroporation, a well-established technique in the field of biomedical research, entails the controlled administration of brief electric pulses to the skin, resulting in the transient formation of pores [10]. These pores are conduits for enhanced drug delivery, enabling more efficient and targeted therapeutic interventions. The potential of mechanical techniques in augmenting skin permeation exhibits promising results. Nevertheless, the practical implementation of these techniques is currently hindered by certain limitations. These limitations include the necessity for specialized equipment and the possibility of inducing skin irritation [12]. Nanotechnology-based approaches, such as nanocarriers (liposomes, polymeric nanoparticles, and nanoemulsions), offer significant advancements in macromolecule administration *via* the dermal route. These carriers protect macromolecules from enzymatic degradation and enhance stability. Their compact size facilitates improved dermal permeation and sustained release, increasing therapeutic efficacy and minimizing unintended effects. Additionally, nanotechnology enables the simultaneous delivery of multiple therapeutics, addressing the limitations of traditional methods and enhancing transdermal transport.

**Fig. 1:**
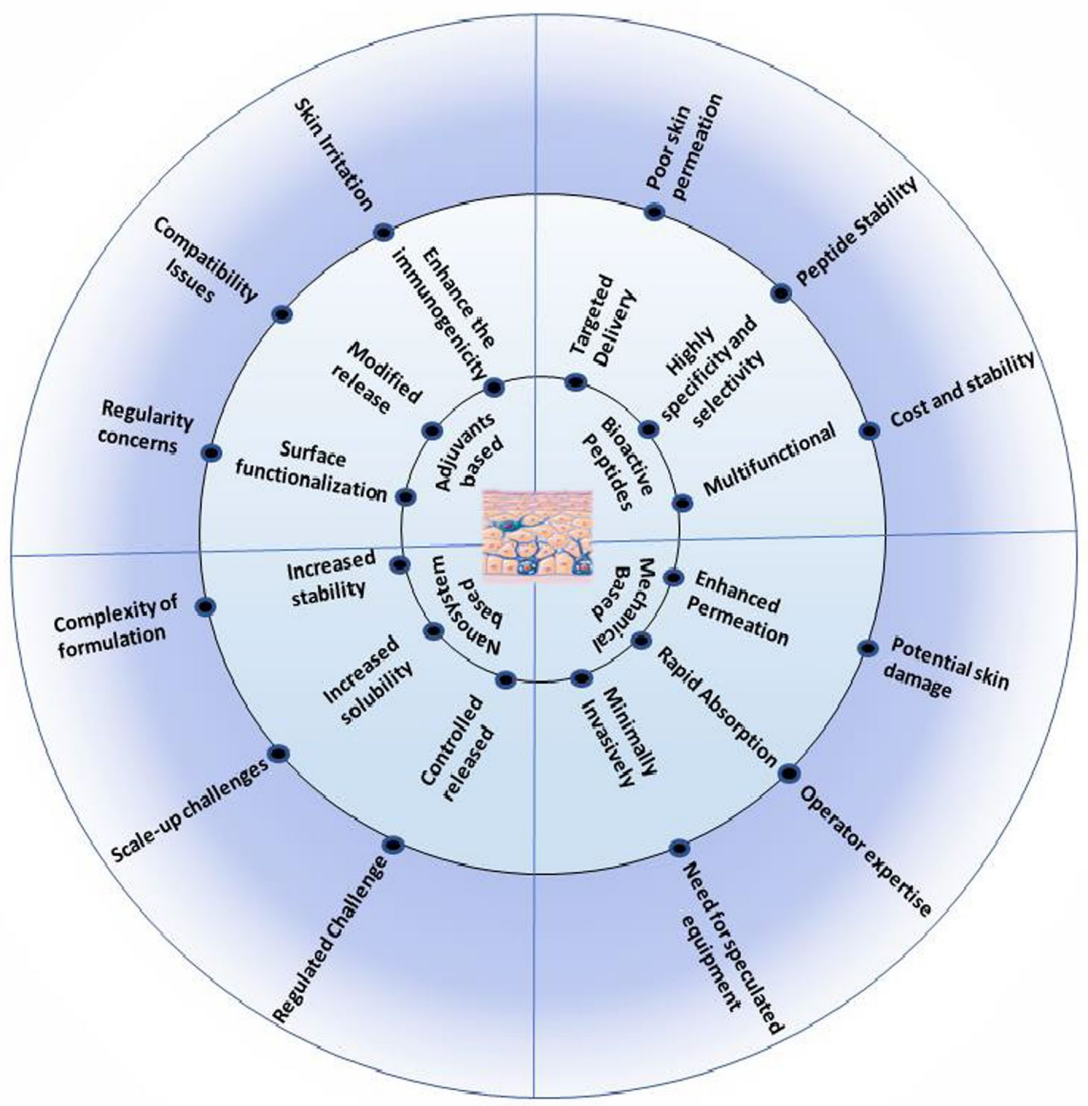
Illustration showing Dermal and Transdermal Macromolecule Delivery: Strategies, Benefits, and Limitations

This study is a comprehensive analysis of the emerging field of macromolecule delivery through the skin, emphasizing the importance of peptides, proteins, and nucleic acids in targeted drug delivery. Although macromolecular therapeutics offer several benefits, they encounter obstacles such as limited skin permeation and vulnerability to degradation. Various conventional and mechanical methodologies have been utilized, although they are not without limitations, such as off-target effects and skin irritation. Nanotechnology-based methods, exemplified by the utilization of nanocarriers, exhibit considerable potential in addressing the constraints by providing augmented stability, precise targeting capabilities, and enhanced skin permeation. Through these advancements, it is possible to attain improved and individualized therapeutic outcomes (Fig. 1).

## The conventional aspect of dermal and transdermal delivery of macromolecules

Conventional therapeutic approaches are paramount in facilitating the transdermal delivery of macromolecules. These approaches encompass various formulations, including gels, creams, ointments, etc. The utilisation of these formulations is crucial in tackling the difficulties related to the transit of large sized and hydrophilic therapeutic agents across the skin barrier, which poses a considerable hindrance in attaining desired concentration of macromolecular therapeutics [13]. Enhancing drug bioavailability is a crucial objective of conventional therapies, aiming to optimise the delivery of the therapeutic agent to the intended site of action to achieve the desired therapeutic effects. In addition to enhancing the bioavailability of drugs, conventional therapies also strive to mitigate systemic toxicity. The systemic administration of large molecules can lead to off-target effects and potentially induce adverse reactions in tissues that are not the intended target [14]. While conventional dermal and transdermal administration techniques generally aid in systemic drug distribution, they may also provide localized benefits in some situations, potentially lowering the possibility of systemic adverse effects [16]. This localised drug release approach enhances the safety and tolerability of treatments for patients. Patients demonstrate a greater probability of complying with their prescribed treatment regimens when said regimens do not necessitate invasive procedures, such as injections or surgeries. The heightened level of compliance can result in enhanced adherence to treatment protocols and ultimately lead to more favourable therapeutic outcomes [17].

Gels are semisolid formulations with shear-thinning properties, making them easy to apply and ensuring rapid drug release upon skin contact. The delivery of hydrophilic macromolecules is effectively facilitated, resulting in enhanced permeation of the skin and reduced greasiness. Nevertheless, gels may require more frequent administrations due to their diminished drug-loading capacity compared to alternative formulations [18]. An innovative local sustained anti-oncogene delivery method was introduced by Yang et al. This method used a PECE thermoresponsive hydrogel and folate-poly (ester amine) (FA-PEA) polymer/DNA complexes. The study on the biodegradable FA-PEA polymer revealed its efficacy in targeted gene delivery. Additionally, the composite could sustain gene release over time when combined with the PECE hydrogel. These findings suggest that this approach holds promise for targeted gene delivery. [19] In another study, Chen et al. synthesized two gelators, namely hyaluronic acid-β-cyclodextrin conjugate (HA-CD) and dextran-2-naphthylacetic acid conjugate (Dex-NAA). The gelators formed the supramolecular hydrogel due to the robust host–guest interaction between β-CD and 2-NAA. The manipulation of the HA-CD and Dex-NAA ratios enabled the regulation of various hydrogel properties, including pore size, gelation time, swelling ratio, and modulus. Overall, the findings suggested that the HA-Dex hydrogel might be used as a cell scaffold in tissue engineering applications [20]**.**

In contrast, ointments are characterised by their greasy and semisolid nature, which allows them to establish a protective layer on the skin, thereby minimising moisture evaporation and facilitating the absorption of medicinal substances [21]. Lipophilic molecules, such as certain hormones (e.g., testosterone) [22], fat-soluble vitamins (e.g., vitamin A & D) [23], can be effectively delivered using ointments. Although ointments can offer prolonged drug release, they can potentially leave behind a tacky residue and may not be universally well-tolerated among patients [24]. In the study conducted by Nakamura et al., the researchers administered delgocitinib ointment 0.5% and several topical corticosteroids (TCSs) to mice over 14 days. Although topical corticosteroids (TCSs) have been associated with adverse effects such as skin thinning and other alterations, applying delgocitinib ointment 0.5% did not induce any of these effects above. According to a study [25], it can be inferred that the utilisation of delgocitinib ointment 0.5% may present a comparatively safer option for the treatment of atopic dermatitis over existing topical corticosteroid (TCS) therapies, particularly in the context of long-term management. In an independent study, Djemaa et al. conducted research demonstrating the significant enhancement of wound healing in rats by applying lavender ointment. This finding suggests that lavender ointment can be utilised as a therapeutic agent for treating skin injuries [26]**.**

Nevertheless, traditional treatments do possess certain constraints. A notable limitation lies in efficiently administering macromolecules having high molecular weights. The skin's inherent protective layer, specifically the outermost layer of the epidermis, stratum corneum, is a barrier that limits the entry of large hydrophilic molecules into the skin [27]. To overcome this constraint, scholars have investigated diverse approaches, including chemical penetration enhancers and mechanical techniques, intended to enhance the permeability of macromolecules across the skin [11]. Another obstacle pertains to the attainment of prolonged drug release over extended durations. Several conventional formulations may not offer a sustained therapeutic effect, requiring more frequent administrations to sustain drug concentrations within the therapeutic range. This constraint can pose significant challenges for individuals with chronic ailments, necessitating extended therapeutic interventions. Furthermore, certain patients can encounter skin irritation or allergies due to the presence of specific ingredients found in conventional formulations. The aforementioned negative consequences can influence patients' adherence and may necessitate the cessation of treatment or the exploration of alternative therapeutic strategies [28].

## Current approaches for dermal/transdermal delivery of macromolecules

Recently, significant advancements have been made in macromolecular dermal and transdermal delivery that seek to improve the penetration of these vital molecules through the skin barrier [29]. One of the categories that can be identified is chemical enhancers, which can enhance the permeability of drugs through the skin. However, using chemical enhancers raises concerns regarding long-term integrity and skin irritation [30]. A study discovered that using laurocapram and its derivatives might induce skin irritation and allergic responses [31]. Similarly, oleic acid may cause skin irritation and dryness [32]. The extended utilisation of specific enhancers can disturb the skin's innate protective layer, resulting in compromised structural stability and increased susceptibility to irritation and sensitivity []. Achieving a harmonious equilibrium between optimal efficacy and the preservation of skin integrity is of utmost importance, thereby necessitating continuous investigation into the development of safer permeation enhancers [33]. For instance, natural terpenes have gained significant attention due to their high enhancement effect and low skin irritation [34] Healthcare professionals aim to discover substances that minimise potential hazards while maximising the effectiveness of transdermal drug administration, thereby maintaining the skin's integrity and durability over extended periods of usage [1]. In addition to using chemical enhancers, researchers have also investigated mechanical approaches to augment the permeability of drugs through the skin [35]. These mechanical techniques use physical forces to overcome the skin barrier and improve macromolecule transport. One method that exhibits potential for transdermal delivery of charged macromolecules is iontophoresis.

Nevertheless, this technique possesses limitations as it can only be applied to specific categories of macromolecules with an electric charge [9]. Moreover, implementing this technique necessitates utilising specialised equipment, thereby diminishing its convenience and accessibility in comparison to alternative modes of delivery [24]. While iontophoresis has advantages, its application in macromolecule delivery is limited by certain constraints [36]. However, the emergence of sophisticated technologies instils a sense of optimism. Microneedles refer to diminutive, needle-shaped structures that generate microchannels within the dermis, thereby enabling the administration of pharmaceutical agents and diverse medical interventions. The needles commonly utilised are fabricated from biocompatible materials and can be either solid, hollow, or dissolvable. Upon application to the skin, microneedles effectively perforate the stratum corneum, which serves as the skin's outermost barrier. This process facilitates the infiltration of macromolecules, medications, or therapeutic agents into the underlying layers of the skin [37]. Microneedles present a range of benefits during the treatment phase, including augmented transdermal drug administration, heightened drug bioavailability, diminished pain and discomfort relative to traditional injections, and potential utility in vaccination, dermatology, and cosmetic interventions [38]. Microneedles possess the advantageous characteristic of circumventing the stratum corneum, thereby enabling painless administration. However, it should be noted that microneedles do not achieve targeted delivery [40]. Therefore, combining it with iontophoresis offers advantages such as precise administration, compatibility with charged compounds, and the ability to regulate release [41]. This technique entails the application of a low-intensity electrical current to aid the transdermal transportation of charged molecules, including ions or pharmaceutical substances [42]. The underlying mechanism of this process is based on the fundamental principle of electrostatic repulsion, wherein similar charges exhibit a mutual force of repulsion [43]. Consequently, this repulsive force propels the charged molecules towards the skin, countering their inherent concentration gradient.

After investigating diverse mechanical approaches for skin delivery, it becomes evident that current methodologies have certain constraints [44]. These limitations encompass the constrained permeability of macromolecules, limited stability of drugs, and inconsistent rates of drug release [45]. Recently, nanotechnology has emerged as a promising approach to tackle these challenges and improve transdermal drug delivery. This field entails the manipulation of matter at the nanoscale to develop novel drug delivery systems. Nanotechnology presents significant advantages in transdermal drug delivery [46]. Nanoparticles offer a means of achieving precise and prolonged drug delivery, thereby resulting in enhanced therapeutic efficacy. This particular characteristic holds significant value for medications that necessitate an extended duration of action, as it diminishes the need for frequent administration and guarantees a steady concentration of the drug in the circulatory system [47]. Several types of nanocarriers, such as liposomes, polymeric nanoparticles, transfersomes, etc, have been extensively investigated for their potential applications in transdermal drug delivery. Liposomes, which are vesicles composed of phospholipids, possess the ability to encapsulate drugs with both hydrophobic and hydrophilic properties. This characteristic renders them highly adaptable as carriers for drug delivery. The distinctive composition of these substances facilitates the adequate transportation of various therapeutic agents across the skin's barrier [48].

In contrast, polymeric nanoparticles present a stable and adaptable framework for encapsulating pharmaceutical agents, facilitating regulated drug release and enhancing drug durability. In pharmaceutical research, transfersomes have emerged as a distinctive variant of liposomes, specifically designed to augment the permeation of substances through the skin. This innovative technology aims to optimise drug administration by enabling deeper penetration and enhancing the efficacy of drug delivery. These carriers possess significant potential in addressing the obstacles related to transdermal drug administration, thereby guaranteeing the most favourable therapeutic results for diverse medical interventions [49].

## Limitations associated dermal/transdermal delivery of macromolecules

**Fig. 2:**
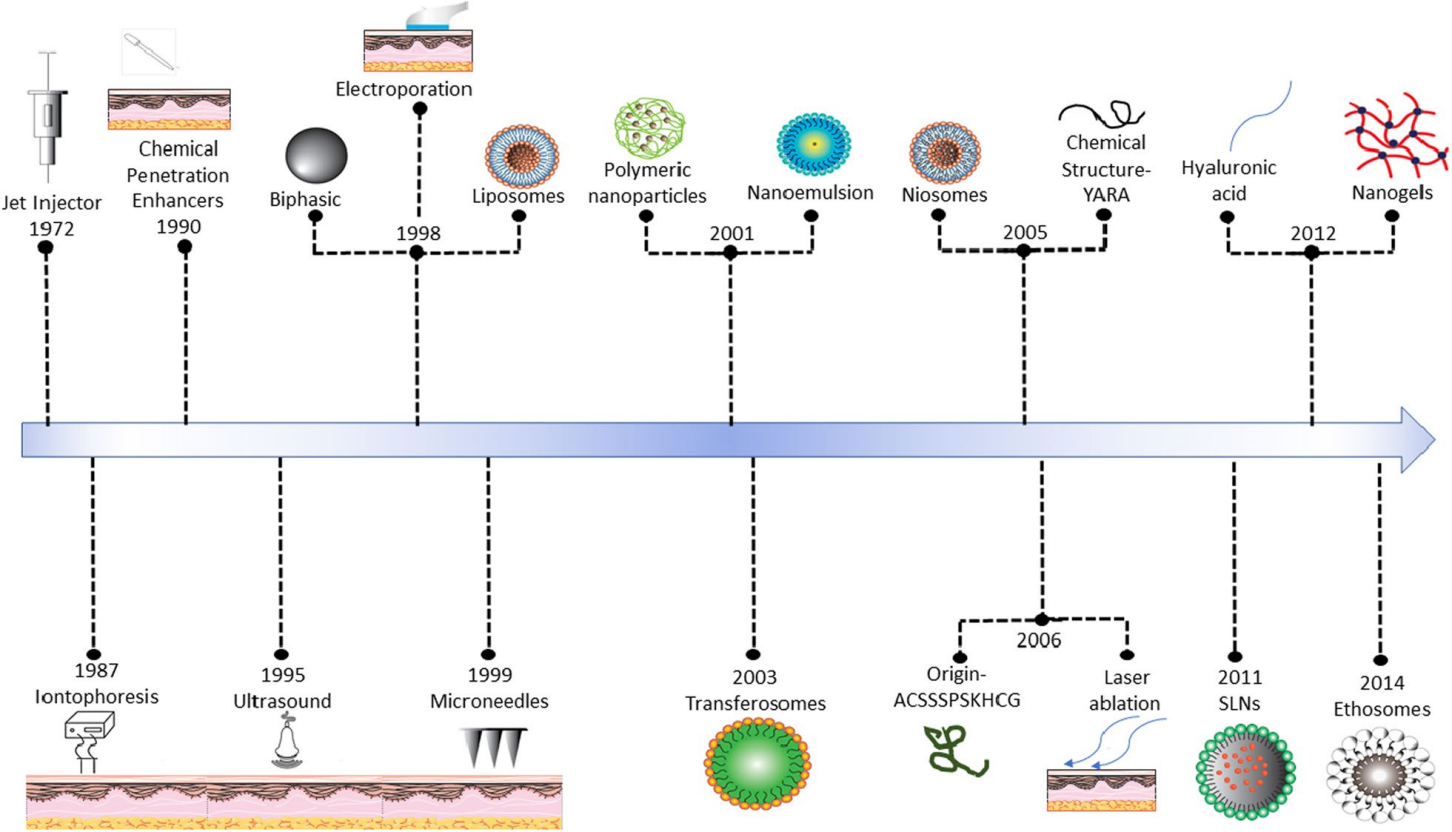
Evolution of Topical Macromolecule Delivery: A Timeline from Jet Injectors to Advanced Nanocarriers [53–71]

As discussed earlier, conventional delivery systems have certain limitations, like the delivery of high molecular weight macromolecules and the permeation of hydrophilic molecules through the natural skin barrier, reducing the therapeutic efficacy. Researchers have been working on various approaches to improve drug permeability into the skin to address this. They aim to disrupt the stratum corneum microstructure and enhance therapeutic delivery through the skin [11]. Despite significant progress, several limitations hinder widespread adoption and efficacy. A primary concern is the stability of macromolecules during storage and delivery [50]. Macromolecules, especially proteins and peptides, are sensitive to environmental factors, and denaturation or degradation can lead to a loss of their therapeutic activity [3]. Therefore, formulating stable and bioactive macromolecules in transdermal delivery systems requires careful consideration of excipients, storage conditions, and manufacturing processes [51]**.** Ensuring the integrity of macromolecules throughout the delivery process is crucial to achieving their desired therapeutic effects. A major challenge is the limited loading capacity of transdermal delivery systems for macromolecules [4]. High doses of large molecules can be impractical and uncomfortable for patients, irrespective of the delivery system used. Therefore, optimizing drug-loading capacity is essential for efficient and effective macromolecule delivery, whether using patches, microneedles, or other methods [52] (Fig. 2, Table 1).

**Table 1:** Strategies for Enhancing Dermal and Transdermal Macromolecule Delivery

Strategy	Sub-variant	Payload Used	Indication	Inference	References
Adjuvants	Chemical Penetration Enhancers	Amino Acids, Dipeptides and Pentapeptide, Enkephalin	–	Leu-Enkephalin diffusion was studied in the presence of 100 mM NDMS, resulting in approximately 26,000 Dpm, while the control had around 6,000 Dpm of Leu-Enkephalin diffusion	[72]
		Insulin	–	Resulted in reduced blood glucose levels and enhanced plasma hormone levels, which attributed to the inactivation of endogenous sulfhydryls	[73]
		Macromolecules (∼1–10 kDa), Heparin, LHRH Oligonucleotides, Leuprolide Acetate	Systemic delivery of macromolecules without skin irritation	Feasibility of using penetration enhancers for systemic delivery of macromolecules from a transdermal patch; demonstrated increased skin permeability and safety in in vitro and in vivo experiments	[74]
		IFNα (19 kDa protein)	Investigation of SC lipid disordering effect on macromolecule permeation	Increased SC lipid disorder caused by surfactants and terpenes, but did not correlate with increased absorption of IFNα	[75]
	Hyaluronic Acid-Based Adjuvants	Bovine Serum Albumin/Hyaluronic Acid	–	The in vitro toxicity studies revealed the absence of toxicity in fibroblast and keratinocyte cells for the range of 0.5–2% v/v formulation	[76]
		TNF-Α Inhibitors	Burn Wound	There was also an observation of enhanced re-epithelialization in conjugate-treated sites, along with reductions in inflammatory markers and secondary tissue necrosis	[77]
Bioactive Peptides	Cell Penetrating Peptides	Short Synthetic Peptide ACSSSPSKHCG With Insulin	Diabetic Wound	The co-administration of peptides also enhanced the systemic levels of human growth hormone by enabling the systemic delivery via the creation of transient openings in the skin	[55]
		Transdermal Peptide (TD)(ACSSSPSKHCG), With Human Growth Hormone (GH)	Alzheimer's Disease	Submicron-sized particles enhance growth hormone (GH) transport across the stratum corneum, enabling it to reach the viable layers of the skin, unlike GH alone, which localizes in the stratum corneum	[78]
		Non-covalently linked PTDs, YARA, Covalently linked PTD, WLR	–	Non-covalently linked PTDs can enhance peptide penetration; covalently linked PTDs show greater efficiency in promoting penetration into viable skin	[79]
		SiRNA, AT1002	–	AT1002 modulates tight junctions to enhance paracellular transport; TAT enhances cell penetration and siRNA stability. Combined use of Tat and AT1002 peptides significantly increases transdermal siRNA delivery efficiency	[80]
Mechanical Methods	Iontophoresis	Insulin Gel	–	The combination of iontophoresis along with linoleic acid showed maximum insulin penetration (411.44 µg) in comparison to periodic iontophoresis (107.62 µg) and the passive delivery of insulin (51.27 µg)	[81]
		Antisense oligonucleotide (ASO)–dendrimer complex	Enhanced delivery to viable epidermis	Electromigration is the dominant mechanism. Iontophoretically delivered ASO–dendrimer complex reduces tumour volume by 45%, lowers Bcl-2 protein levels, and induces significant apoptosis in skin tumours	[82]
		STAT3 siRNA	Melanoma treatment	AuNP is coated with chitosan (AuNP-CS) and layered with siRNA and chitosan. Significant cell growth inhibition and uptake via clathrin-mediated endocytosis. Reduces STAT3 protein expression and induces apoptosis in melanoma cells. Anodal iontophoresis enhances skin penetration to reach the viable epidermis	[83]
		Cx43 specific antisense oligodeoxynucleotide	Enhanced drug permeation	Cathodal iontophoresis increased drug permeation threefold. Combining both approaches resulted in a fourfold enhancement in Cx43 specific antisense oligodeoxynucleotide permeation. Complete permeation not achieved; molecules trapped in epidermis or permeated deeply into hair follicles	[84]
		LHRH	Increased drug permeability through the human epidermal membrane	Combining 5% oleic acid in propylene glycol with iontophoresis resulted in a 29.5-fold increase in permeability. Only iontophoresis showed a 14.3-fold increase, and only the chemical penetration enhancer exhibited a 3.5-fold increase	[85]
	Ultrasound-Assisted Delivery	Insulin	Diabetes	Ultrasound-assisted insulin treatment effectively decreased blood glucose levels in Yorkshire pigs by up to −91 ± 23 mg/dL within 90 min compared to the control group	[86]
		Radiolabeled oligonucleotides	–	Ultrasound-assisted application for 10 min resulted in oligonucleotide concentrations in superficial skin layers and the formation of localized transport pathways	[87]
		Insulin	–	Encapsulated insulin released rapidly upon ultrasound treatment, effectively regulating blood glucose levels	[88]
		Liquid-type EGF-coated lysozyme microbubble (LYMB) cavitation	Wound healing	Significant improvement in recovery rate during the first 6 days compared to control when EGF-LYMB dressings were used with ultrasound exposure	[89]
	Needle-Free Jet Injection	DNA Vaccine	–	The delivery of dna vaccine and observed that the combination was able to multiply the rate and magnitude of dna vaccine-induced humoral and cellular responses	[39]
		Influenza vaccines	–	Effective intradermal vaccine delivery with minimal increase in IL6 and MCP-1 levels, demonstrating protective efficacy against influenza	[90]
		Hepatitis B surface antigen (HBsAg)	–	Comparable immune responses to subcutaneous injection with minor bleeding observed at the injection site	[91]
		Human dermal fibroblasts exosomes	Dermal collagen deposition	Increased procollagen type I expression, decreased MMP-1 expression, and higher dermal collagen deposition	[92]
	Temporary Pressure	Insulin	Diabetes	Diabetic mice were able to reduce their blood glucose levels by up to 80% by insulin delivery, with a 1.5 fold increase in the penetration	[93]
	Microneedles	Immunoglobulin Gamma (Igg), Immunoglobulin Gamma-1 (Igg1), And Immunoglobulin Gamma-2α (Igg2α)	–	A significantly lower bacterial skin penetration was observed after zmn application compared with hypodermic syringe application	[94]
		Peanut Protein Extract (PE)	Allergy desensitization	Significantly lower clinical symptoms of peanut-induced anaphylaxis and down-regulation of anaphylaxis mediators	[95]
		SEB protein	Immunization against SEB toxin	Enhanced in vivo retention time of antigens and 100% protection against lethal SEB toxin challenge	[96]
		Plasmid DNA	Gene therapy	High transfection efficiency and sustained release profile in vitro and in vivo	[97]
	Laser-Assisted Delivery	SiRNA	–	In vitro experiments indicated a 2.4- 10.2-fold increase in sirna permeation with laser exposure as compared with the nontreated group	[98]
		Hexameric Insulin, FITC-labelled dextran	–	Enhanced delivery without altering viable skin morphology; facilitated transdermal delivery of large molecules	[99]
		hGH, FSH, FITC-labelled BSA	–	Increased permeation of hGH, FSH, and FITC-BSA compared to control	[100]
	Electroporation	Insulin	Diabetes	The decrease in applied field strength (from 200 to 100 v/cm) resulted in a significant decrease in blood sugar levels compared with control	[101]
Nanotechnology based Methods	Nanoemulsion	Epidermal Growth Factor (EGF), Insulin-Like Growth Factor-I (IGF-I), And Platelet-Derived Growth Factor-A (PDGF-A)	Burn Wound	Incorporating nanoemulsion dispersed hydrogel led to increased fibroblast proliferation, improved scratch wound healing, and enhanced skin permeation of active ingredients	[102]
		Salmonella enterica outer membrane antigens	Topical vaccine administration	A nanoemulsion formulation for topical vaccine delivery using outer membrane vesicles, inducing specific antibody response	[103]
	Solid Lipid Nanoparticles	Tristearin Solid Lipid Nanoparticles Loaded With Sirna	–	The formulation was able to provide a prolonged sirna release over 10–13 days, along with the retention of the activity of the released SiRna, as indicated by the in vitro studies	[104]
		Ovalbumin	Enhanced delivery through hair follicles	Efficient penetration and cellular response of OVA-loaded PLA nanoparticles through hair follicles	[105]
		Amorphous heptapeptide DEETGEF	Activation of cell-protecting enzymes	Significant activation of protective enzymes with SLNs loaded with amorphous heptapeptide DEETGEF	[106]
	Liposomes	Low-Molecular-Weight Heparin (LMWH	Thrombosis, Subcutaneous Wounds, Bruise, And Burns	Better physicochemical stability than the other formulations, along with enhancing the in vitro skin penetration and in vivo localization into the deeper skin layers	[107]
		Recombinant human TG1	Treatment for autosomal-recessive congenital ichthyosis	Effectively delivered rhTG1 to keratinocytes using sterically stabilized liposomes, improving skin barrier function	[108]
		siRNA	Gene silencing, topical delivery	Demonstrated effective topical gene silencing using liposomal carriers, targeting psoriasis markers	[109]
	Niosomes	DNA Encoding Hepatitis B Surface Antigen (Hbsag)	–	The formulation was able to elicit a comparable serum antibody titer and endogenous cytokines levels compared to intramuscular recombinant Hbsag	[70]
		Serratiopeptidase	Topical anti-inflammatory activity	Increased steady-state flux and significant permeation enhancement	[110]
	Ethosomes	SiRna	–	The combination of space peptides resulted in a knockdown of 83.3 ± 3.0% compared to the control, demonstrating effective delivery of siRNA in female BALB/C mice	[71]
		Palmitoyl pentapeptide	–	Improved permeation through artificial and human skin	[111]
		Thymosin β−4	Wound healing	Reduced wound healing time in in vivo studies	[112]

## Adjuvants

Adjuvants play a crucial role in delivering macromolecules through the skin. The skin barrier poses challenges for effectively delivering large molecular-weight drugs like proteins, peptides, and nucleic acids [113]. Adjuvants, while traditionally recognized for their role in enhancing drug delivery, also exhibit significant immunological functions. Beyond facilitating dermal and transdermal macromolecule permeation, adjuvants can modulate immune responses, such as enhancing antigen presentation or activating immune cells at the site of application. This dual functionality underscores their potential not only as delivery enhancers but also as immunomodulatory agents, particularly in applications like vaccine formulations. Adjuvants come to the rescue by aiding in overcoming this obstacle, thereby enhancing the permeation of these therapeutic agents and opening up new possibilities for transdermal drug delivery [114]. Two major categories of adjuvants are commonly discussed: plant-based and chemical-based adjuvants. Plant-based adjuvants utilize natural compounds derived from plants, such as terpenes, flavonoids, and essential oils, which have promising penetration-enhancing properties [115]. On the other hand, chemical-based adjuvants consist of various synthetic compounds that also exhibit the ability to promote drug permeation through the skin [15]**.** Both categories offer distinct advantages and are currently under extensive exploration to optimize transdermal drug delivery for various therapeutic applications.

### Plants-based adjuvants

Plant extracts have emerged as promising candidates, offering biocompatibility, safety, and targeted delivery improvement [116]. However, a major challenge in their topical delivery is their limited ability to penetrate the skin effectively. Plant extracts have demonstrated potential as penetration enhancers, facilitating the transport of macromolecules across the skin [117]. Compounds such as terpenes [118], flavonoids [119], and essential oils [120] found in plants disrupt the skin's lipid structure, leading to the formation of transient aqueous pores. This process enhances macromolecule diffusion into deeper skin layers, significantly improving drug absorption and bioavailability [11].

Furthermore, plant-based adjuvants offer a sustainable and biodegradable alternative to the synthetic carriers commonly employed in drug delivery. For instance, Songkro et al. conducted a study to investigat the effect of oxygen-containing terpenes (carvacrol, menthol, and carvone) at 5% *w/v* in hydroalcoholic mixtures on the permeation of luteinizing hormone-releasing hormone (LHRH) across newborn pig skin in vitro. They also determined the amount of LHRH retained in the skin after 24 h of diffusion. The application of terpenes significantly increased the percutaneous absorption of LHRH, with the highest effect observed for carvacrol, followed by carvone and menthol. Additionally, terpenes enhanced the retention of LHRH, with carvone showing the most substantial enhancement [118]. Hence, plant-based adjuvants show great promise in overcoming the challenges of large molecular therapeutics and skin penetration. Their ability to improve drug delivery efficiency while offering sustainability makes them a valuable option for future pharmaceutical advancements.

### Chemical penetration enhancers

**Fig. 3:**
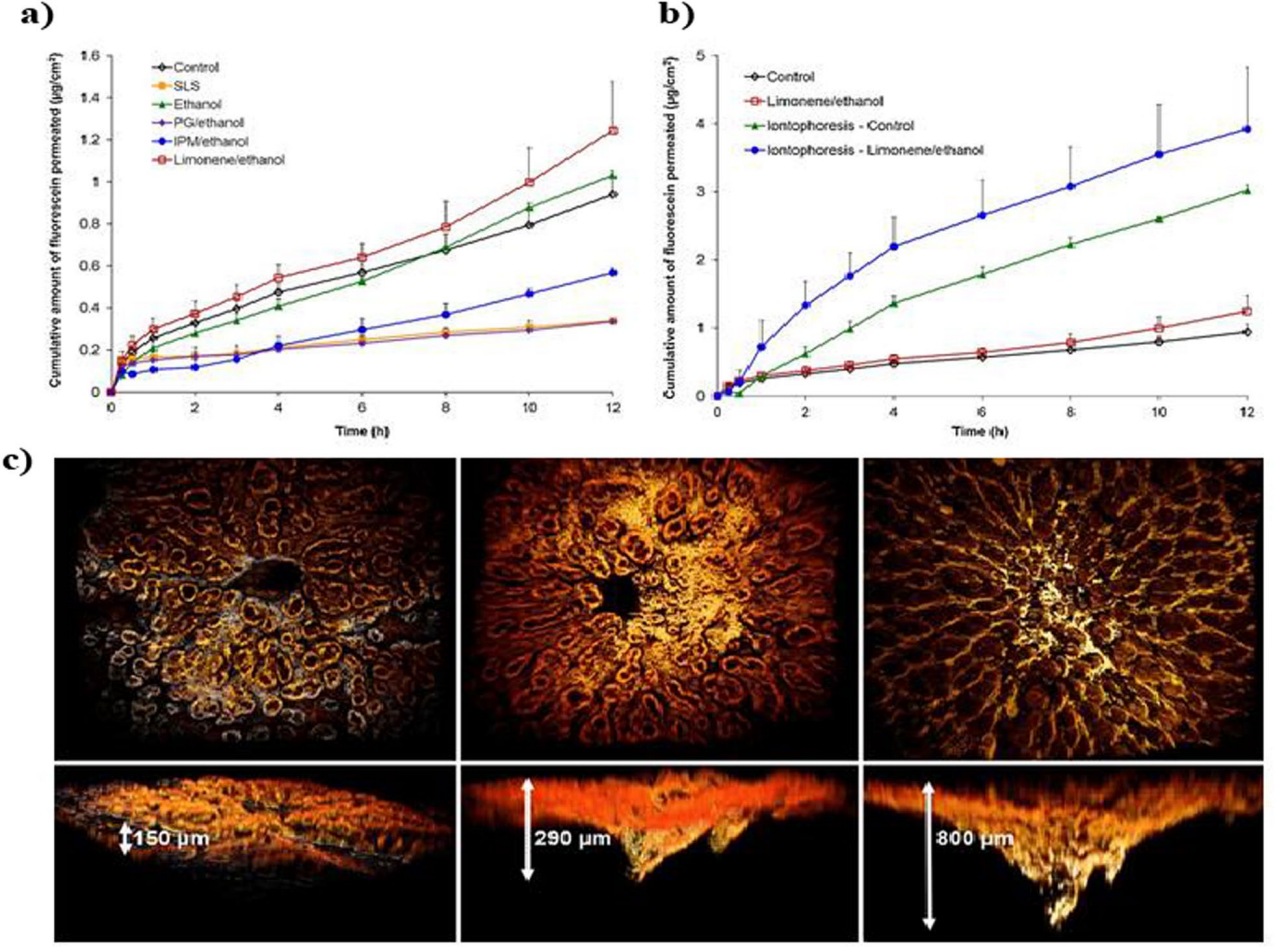
**a**, **b** Cumulative amount of sodium fluorescein permeated across untreated and enhancer pretreated porcine skin, and with and without iontophoresis versus time; **c** skin tissue after 12 h of Cx43 AsODN permeation with the top row showing the skin surface with the keratinocyte pattern and a hair follicle in the centre of the image and the bottom row illustrating the side-on view demonstrating the penetration depth of the Cy3-tagged AsODN. Reprinted with permission from [84]

Chemical penetration enhancers are facilitators for transporting therapeutics through the skin's barrier, particularly the stratum corneum, by temporarily altering the skin's structure by disrupting the lipid bilayers or increasing skin hydration [6]**.**  Consequently, this reduces barrier resistance, allowing improved therapeutic penetration, which offers multiple benefits, including non-invasiveness, easy application, and improved patient compliance [35]. However, it is crucial to consider the safety and potential irritation caused by some penetration enhancers. Selecting and optimizing the formulation carefully becomes necessary to prevent skin irritation or allergic reactions. Additionally, monitoring the risk of systemic drug absorption is essential to avoid unintended adverse effects [121] (Fig. 3).

The effects of chemical penetration enhancers have been studied for the delivery macromolecules. For example, Choi et al. investigated the impact of n-decyl methyl sulfoxide (NDMS) on the permeation of amino acids, dipeptides, and the pentapeptide enkephalin through hairless mouse skin. They observed that NDMS substantially improved the permeation of all tested peptides. Leu-enkephalin's diffusion, in the presence of 100 mM NDMS, was approximately 26,000 disintegrations per minute (dpm), whereas the control formulation allowed only around 6000 dpm of Leu-enkephalin to diffuse through the cells [72]. Similarly, Sintov et al. investigated the impact of skin pretreatment with iodine followed by insulin application which reduced blood glucose levels and increased plasma hormone levels, attributed to inactivating endogenous sulfhydryls like glutathione and gamma-glutamylcysteine, thus safeguarding the dermally administered insulin [73]. Continued exploration of novel approaches to enhance skin permeability has focused on investigating combinations of penetration enhancers and their effects on macromolecule absorption and stratum corneum barrier lipids. For example, Karande et al. investigated homogeneous mixtures of penetration enhancers to enhance skin permeability to macromolecules (∼1–10 kDa) by approximately ∼100-fold without causing skin irritation. In vitro experiments evaluated the impact of these chemical enhancers on macromolecules such as heparin, LHRH, and oligonucleotides. In vivo studies on hairless rats validated the effectiveness and safety of a specific mixture containing sodium laureth sulfate (SLS) and phenyl piperazine (PP) [74]**.** Similarly, Moghadam et al. investigated the impact of chemical permeation enhancers on stratum corneum barrier lipids' organisational structure and interferon-alpha (IFNα) permeability. They tested various agents, including solvents (ethanol, propylene glycol, diethylene glycol monoethyl ether—transcutol, oleic acid), terpenes (menthol, nerol, camphor, methyl salicylate), and surfactants (Tween 80, SDS, benzalkonium chloride, polyoxyl 40 hydrogenated castor oil—Cremophor RH40, didecyldimethylammonium bromide—DDAB, didecyltrimethylammonium bromide—DTAB). The study found that these agents caused disordering of the lamellar and lateral packing of lipids. However, the in vitro permeation studies revealed that the increased disordering of lipids had minimal influence on the absorption of IFNα [75]. Hence, relying solely on chemical enhancers can be insufficient in effectively increasing the skin permeability of macromolecules. Several studies have explored a combination approach to enhance the skin permeability of therapeutic macromolecules to address this limitation.

Among the chemical penetration enhancers, a prominent category includes adjuvants based on Hyaluronic acid (HA), a polysaccharide composed of alternating monomer units of N-acetyl glucosamine and glucuronic acid, with an average molecular weight of 2 × 10^5^ to 1 × 10^7^ Da [122]. HA exhibits remarkable penetration enhancer properties, making it an ideal candidate for skin delivery [123]. Its unique capacity to retain up to 1000 times its weight in water enables deep skin hydration and improved drug permeation [123]. By enhancing skin penetration, HA facilitates better absorption of active ingredients, leading to enhanced efficacy and therapeutic benefits in skincare products [124]. For instance, Martins et al. worked on the development of Bovine Serum albumin/Hyaluronic acid (BSA/HA) nanodispersions for enhancing the permeability of BSA via the transdermal route by conjugating and converting it into solid-in-oil (S/O) nanodispersion with a mean diameter of 129.7 nm. Ex vivo skin penetration analysis by fluorescence and confocal observation of histological skin sections revealed the ability of BSA/HA nanodispersions to cross the stratum corneum and penetrate the dermis [76]. In another research, Witting et al. explored the impact of HA hydrogels with different molecular weights on the skin absorption of bovine serum albumin (BSA) using fluorescence lifetime imaging microscopy (FLIM) and Fourier-transform infrared (FTIR) spectroscopy. They observed that HA hydrogels limited BSA penetration into the epidermis in barrier-deficient skin, whereas low molecular weight HA facilitated BSA penetration in normal skin. Furthermore, HA influenced stratum corneum constituents, causing a α-helix to β-sheet interconversion of keratin and increased skin hydration. These findings highlight HA hydrogels as potential topical drug delivery systems capable of modulating biomacromolecule transport in the skin [125]**.** Further, to investigate the conjugation and complexation of therapeutic agents with HA to enhance wound healing, emphasizing the efficacy of these formulations in promoting skin permeation and improving therapeutic outcomes. For example, Choi et al. conducted a study where they genetically fused low-molecular-weight protamine (LMWP) with growth factors (GFs) and formed a complex with hyaluronic acid (HA). The resulting LMWP-fused GFs-HA complex demonstrated enhanced fibroblast proliferation and wound healing, benefiting from the synergistic properties of HA. To further improve skin permeation compared to native GFs, the complex was incorporated into cationic elastic liposomes (ELs). In a diabetic mouse model, cationic ELs containing the LMWP-fused GFs-HA complex significantly accelerated wound closure, highlighting its potential for promoting the healing of chronic wounds [126]**.** Similarly, Friedrich et al. investigated the healing effects of TNF-α inhibitors with hyaluronic acid in a rat burn model via conjugating anti-TNF-α to high molecular weight HA ([anti-TNFα]-HA), which maintained its binding affinity for TNF-α and potentially reduced antibody transport at the application site. In vivo administration of the free antibody demonstrated its ability to slow down macrophage infiltration in the periphery but not at the surface. In contrast, the conjugated antibody effectively inhibited macrophage infiltration at the edge and the surface, suggesting deeper penetration of the conjugated antibody, potentially enhancing its distribution and therapeutic impact within the targeted tissue [77].

Yang et al. explored a receptor-mediated transdermal delivery method using a transdermal hyaluronic acid-human growth hormone conjugate (HA-hGH). They utilized HA receptors in keratinocytes and hGH receptors in fibroblasts. The biological activity of the conjugate was confirmed in fibroblasts through increased expression of phosphorylated Janus kinase 2 (p-JAK2). Effective skin penetration of the Hyaluronic acid-human Growth Hormone-Fluorescein isothiocyanate (HA-hGH-FITC) conjugate was observed using fluorescence microscopy after topical application. Pharmacokinetic analysis indicated that the HA-hGH conjugate likely entered the bloodstream via receptor-mediated transdermal delivery, demonstrating its potential as a promising approach for delivering therapeutic agents through the skin [54].

## Bioactive peptides

Bioactive peptides present promising opportunities for transdermal drug delivery due to their ability to penetrate the skin’s stratum corneum (SC) efficiently[127]. Bioactive peptides can be categorized based on their origin, including protein-derived CPPs, model peptides, chimeric CPPs, and synthetic CPPs. Additionally, they can be classified by their chemical structure into amphipathic CPPs, cationic CPPs, and hydrophobic CPPs. These bioactive peptides play a crucial role in overcoming the SC barrier and have the potential to revolutionize the field of topical macromolecular administration.

### Based on origin

**Fig. 4:**
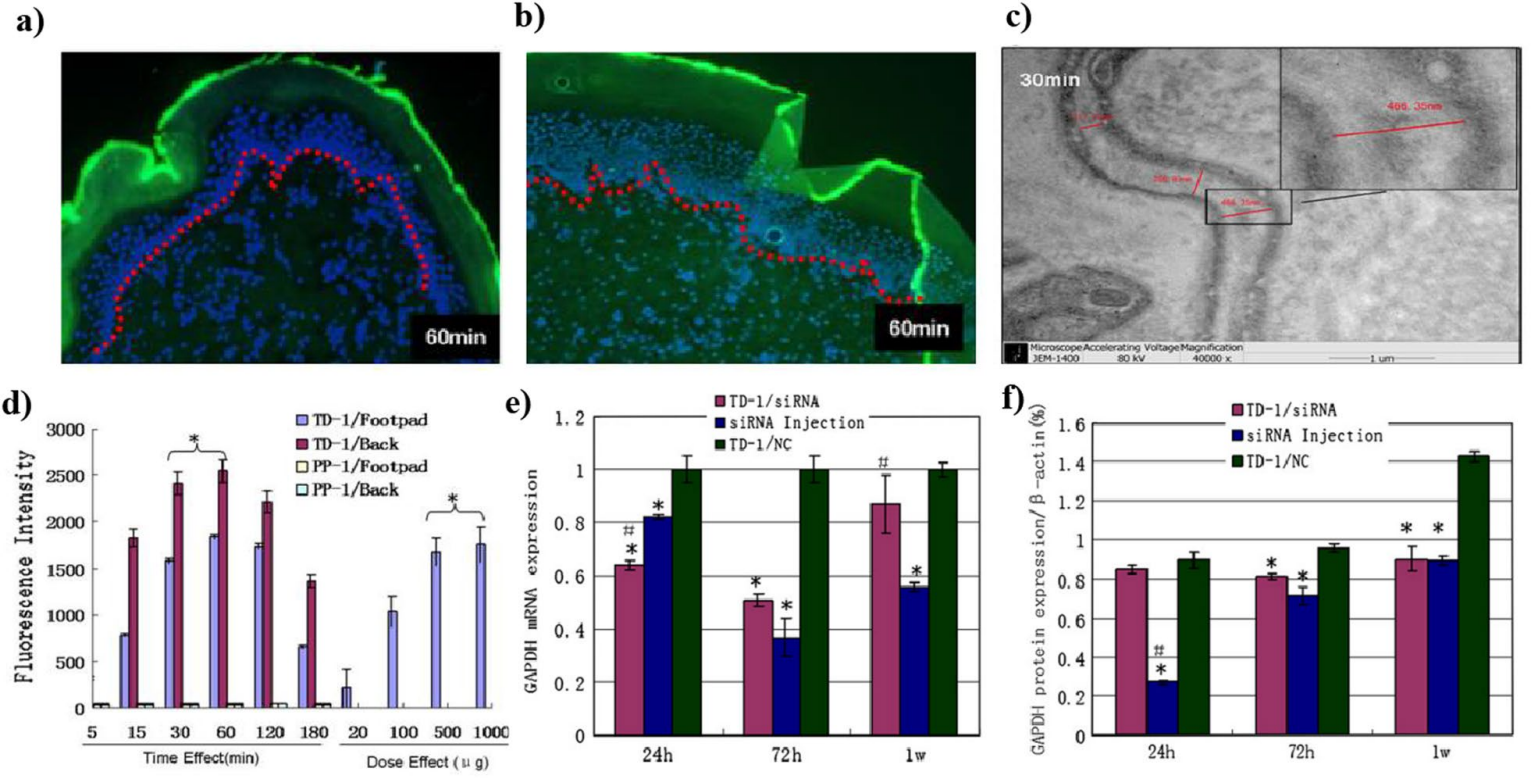
**a** FITC-labelled TD-1 and **b** co-administered FAM-labelled siRNA were detected strongly from epithelial tissue to subcutaneous tissue 60 min after application; **c** Transmission electron microscopy of epithelial cells enlarged spaces between cells after 30-min topical application of TD-1); **d** Time and dose effect of TD-1 topically applied to the footpad and back skin of the rat; **e**, **f** mRNA and protein expression of GAPDH by TD-1 transdermal delivery or intradermal injection of anti-GAPDH siRNA measured by real-time PCR and western blot analysis. Reprinted with permission from [132]

CPPs can be classified into four categories based on their origin and composition viz. Protein-derived CPPs, Model Peptides, Chimeric CPPs, and Synthetic CPPs. Protein-derived CPPs, such as TAT and Antennapedia peptides, are derived from natural proteins. Model peptides such as Penetratin and pVEC serve as research tools for studying CPP mechanisms [128]. Chimeric CPPs, exemplified by PepFect and Transportan, combine different CPP sequences or include additional functional motifs. Synthetic CPPs, including Polyarginine and Polylysine, are artificial and commonly consist of repetitive amino acid sequences [129]**.** Protein-derived CPPs originate from natural proteins and can traverse cell membranes, making them valuable for enhancing transdermal drug delivery and intracellular targeting. Among them are the Transactivating transcriptional activator (TAT) (47–57) fragment, originating from HIV-1 trans-activating protein (HIV-1 TAT protein), and Penetratin derived from Antennapedia homeodomain peptides. Model peptides are designed to emulate the structure of existing natural peptides. The next category, chimeric peptides, combines hydrophobic and hydrophilic peptide fragments from different origins, such as Transportation 27 (TP27), a 27-amino acid molecule derived from galanin neuropeptide and mastoparan peptide (wasp venom). These peptides enhance transdermal drug delivery by promoting effective skin penetration and facilitating the delivery of therapeutic agents to targeted sites within the skin [130]. Synthetic CPPs are the ones that are derived from the polyarginine peptide family [131]. From a research perspective, synthetic CPPs have been extensively studied. For example, Chen et al. co-administered a short synthetic peptide (ACSSSPSKHCG) with insulin. This combination increased systemic insulin levels and suppressed serum glucose levels for at least 11 h. Additionally, the peptide co-administration facilitated the penetration of insulin into hair follicles at a depth beyond 600 µm. Furthermore, the co-administration of peptides also enhanced the systemic levels of human growth hormone, enabling systemic delivery by creating transient openings in the skin [55]. Zhang et al. conducted a study wherein the co-administration of a synthetic peptide, Transdermal peptide (TD) (ACSSSPSKHCG), with human growth hormone (GH), significantly enhanced the transport of GH across the stratum corneum, allowing it to reach the epidermis and dermis, as opposed to its localization primarily in the stratum corneum when administered alone [78]. Lin et al. observed that the short synthetic peptide ACSSSPSKHCG (TD-1) led to a uniform distribution of the administered siRNA from the epidermis to the subcutaneous tissue of rat footpad skin. This effect was achieved by creating transient openings in non-follicle rat skin (Fig. 4) [132]. Ruan et al. formulated a combination of Human epidermal growth factor (hEGF) with TD-1, which effectively enhanced the transdermal activity of hEGF. Within 5 min, the combination facilitated the transfer of hEGF into the skin, offering a promising advancement in medical and cosmetic fields [133].

### Based on the chemical structure

Cell-penetrating peptides (CPPs) have become indispensable tools in transdermal drug delivery due to their remarkable capacity to enhance the penetration of therapeutic agents through the skin. Based on their chemical structure, CPPs can be categorized into three types: amphipathic CPPs, cationic CPPs, and hydrophobic CPPs. Each category possesses unique properties that enable efficient traversal of the skin's barrier, facilitating the transport of various bioactive molecules and drugs into deeper skin layers [129]. The amphipathic CPPs are further divided into primary and secondary amphipathic CPPs. Primary amphipathic CPPs are chimeric peptides formed by covalently linking a hydrophobic domain to the nuclear localization signal (NLS), enhancing membrane targeting efficiency. MPG (GALFLGWLFAAGSTMGAPKKKRKV) is an example of an amphipathic CPP, consisting of a hydrophobic domain derived from the fusion sequence of the HIV glycoprotein 41 and based on the Simian Vacuolating Virus 40 Nuclear localization signal (SV40 NLS)- PLLRKW. WSQP is the linker separating the hydrophobic domain from the Nuclear Localization Signal (NLS) [134]. Other examples of this class from natural origin include peptide vascular endothelial-cadherin (pVEC) [135], alternative reading frame (ARF) (1–22) [136], BPrPr (1–28) [137]. The peptide comprises only a few amino acid residues for secondary amphipathic molecules. These peptides interact with the cell membrane phospholipids, forming an α-helix or β-helix structure, activating their amphipathic characteristics [138]. The common examples from this class include Penetratin [139], pVEC [140], and M918 [141]**.** The next category consists of hydrophobic peptides, primarily formed of apolar residues. These peptides are the derivatives of the hydrophobic sequences from proteins that naturally interact with the cell membrane. These include peptides like hepatitis B virus translocation motif [142], integrin b3-fragment [143] or calcitonin fragment [144].

Cationic CPPs typically contain multiple arginine-rich regions or acidic amino acid residues, giving the molecule a positive charge. These peptides are often likened to Trojan Horses in drug delivery systems due to their ability to penetrate cells without eliciting a cellular response. Studies have shown that a minimum of eight positive charges is necessary to ensure efficient uptake of cationic CPPs into the cells [138]. Common examples of this category include HIV-1 Tat, penetratin, Herpes Simplex Virus-1 (HSV-1) VP22 peptide transduction domains, nonarginine (R9), Retinoid X receptor (RXR) 4, etc. [145–149]. Most cell-penetrating peptides are cationic and have been extensively investigated for their capacity to enhance the permeation of therapeutic substances into the skin. A study by Lopes et al. concluded that the protein transduction domain (PTD), YARA, could efficiently carry a conjugated protein P20 across the skin [79]. Lee et al. observed that the conjugation of IFN-γ with penetratin resulted in the penetration of IFN-γ into the epidermis and dermis, compared to control IFN-γ, which could not transduce into the skin [150]. Uchida et al. concluded that combining siRNA with AT1002 improved the siRNA's stability against RNAses, and it reduced the expression of the tight junction protein zonula occludens protein 1 (ZO-1). Moreover, transdermal delivery of the siRNA was significantly enhanced, resulting in higher and deeper localization in the skin than the naked siRNA [80]. Chang et al. developed a series of cationic cyclopeptides based on the sequence of TD-1 to enhance the transdermal delivery of insulin. TD-34 exhibited the highest transdermal activity, effectively reducing blood glucose to approximately 26% of the initial levels [151]. Ookubo et al. observed that the combination of 11-arginine with Peptide No. 10 (an 8 amino acid sequence) found in gliadin protein (a peptide within gluten-containing foods) led to a skin-whitening effect after daily repetitive topical application for two weeks. This suggests that 11-arginine could be potentially utilized as a delivery system for the topical delivery of peptide cosmetics [152].

### Based on the interaction between CPPs and the therapeutic agents

Cell-penetrating peptides (CPPs) can be classified based on their interaction with therapeutic agents: covalent bonding or non-covalent interactions. Covalent interactions involve direct chemical linkage, creating stable conjugates, while non-covalent interactions rely on reversible bonds like electrostatic interactions, providing versatile approaches for intracellular drug delivery. The first category involves CPPs that form a covalent linkage with the therapeutic moiety. This group includes well-known peptides like TAT, penetratin, polyarginine peptide Arg8, Transportan, Virus protein 22 (VP22) from Herpes Simplex Virus (HSV), as well as antimicrobial peptides Buforin I, SynB, and polyproline sweet arrow peptide [153–155]. On the other hand, the second category consists of CPPs that establish non-covalent interactions with the therapeutic molecule. These CPPs function as amphipathic peptide carriers, possessing both hydrophobic and hydrophilic domains, enabling interaction in primary or secondary structures. Direct amphipathic peptides within this category display a sequential arrangement of hydrophobic and hydrophilic residues, whereas secondary amphipathic peptides exhibit a conformational state in which hydrophobic and hydrophilic residues are positioned on opposite sides of the molecule. Notable examples in this group include primary amphipathic peptides like Pep-1 and MPG. These peptides can form stable complexes through non-covalent electrostatic and hydrophobic interactions with oligonucleotides or protein/peptide cargoes, showcasing their potential as efficient carriers for intracellular delivery. Understanding CPP interactions and structural properties is essential for optimizing transdermal drug delivery and improving therapeutic efficacy [156]. Multiple studies have explored using covalently and non-covalently bonded peptides to enhance the transdermal delivery of macromolecules. In a study by Chen et al., it was observed that the combination of Epidermal Growth Factor (EGF) with CPPs facilitated the delivery of EGF across the stratum corneum (SC) layer and into the viable epidermis and dermis. The control EGF showed only slight penetration into the upper granulating layers. Among the tested CPPs, Pep-1 demonstrated the most effective activity [157]. A study by Lopes et al. found that the non-covalent linkage with YARA increased the retention of model peptide P20 in the stratum corneum (SC) by 2.33-fold, while WLR enhanced the penetration by 2.88-fold. Moreover, the study also concluded that the covalently attached molecules demonstrated a greater capacity to improve the peptides' skin permeability thanhe non-covalent molecules [79]. Hou et al. (2007) observed that the skin treated with SR9 (synthetic nonarginine) plus green fluorescent protein (GFP), Trans-Activator of Transcription—Protein Transduction Domain (Tat-PTD) plus GFP, R9 (nonarginine) plus GFP, or R9Z plus GFP mixtures exhibited strong fluorescence in the epidermis, dermis, panniculus adiposus, and even hypodermis, in contrast to the skin treated with only the peptide, where fluorescence was observed solely in the epidermis [57].

## Mechanical force-based enhancements and mechanisms

Mechanical-based methods play a crucial role in enhancing the topical delivery of macromolecules, overcoming the challenges posed by the skin barrier. These methods include iontophoresis, ultrasound-assisted delivery, needle-free jet injection, microneedles, laser-assisted delivery, and electroporation [1, 158].

### Iontophoresis

Iontophoresis is a non-invasive technique used to enhance the penetration of drug molecules through the skin. It applies a small, physiologically tolerable electric current to push drug molecules into the skin. This process increases the delivery and absorption of drugs, making it an effective method for topical drug administration. [36]. Iontophoresis enhances transdermal drug transport via two mechanisms: electrorepulsion and electroosmosis. Electroreplusion is the process by which charged molecules are repelled by electrodes of the same polarity, with repulsion responsible for the charged molecule's travel through the skin. Electroosmosis involves the movement of water from the anode to the cathode under an applied electric current, influencing the transport of ions such as sodium (Na⁺) and chloride (Cl⁻) in the skin. The skin's negative charge promotes the movement of positively charged water, resulting in a bulk motion from anode to cathode. This process aids in moving tiny amounts of uncharged water over the skin [159]. The mechanism of permeation enhancement in iontophoretic delivery can be helpful in modulating therapy, as the termination of electric current results in a rapid decline in systemic drug levels. Ideal peptide candidates for iontophoretic delivery have a high charge-to-mass ratio and a smaller molecular volume. The iontophoretic mobility of a peptide is determined by its secondary and tertiary structure, along with the charge on the peptide, which facilitates the transport of the molecule across the skin during iontophoresis. Moreover, the pH also influences the peptides' mobility, with isoelectric points (pI) below 4 and above 7.4 being the ideal candidates for iontophoretic delivery [81]. Pillai et al. demonstrated that the application of insulin gel along with iontophoresis was able to bring down the plasma glucose levels by 36–40%, followed by the combination of iontophoresis along with linoleic acid showed maximum insulin penetration (411.44 µg) in comparison to periodic iontophoresis (107.62 µg) and the passive delivery of insulin (51.27 µg) [81]. In a study by Kigasawa et al., transdermal iontophoresis efficiently delivered immunostimulatory CpG-ODN into the skin, promoting a systemic immune response and antitumor activity against B16F1 melanoma in mice, and CpG-ODN delivery to the epidermis and dermis, inducing the expression of proinflammatory and Th1-type cytokines. [160]**.**

Electromigration was identified as the dominant mechanism behind the iontophoretic permeation of peptide dendrimers across human skin [161]. In a study, Venuganti et al. observed that the iontophoretically delivered Antisense oligonucleotide–dendrimer complex was able to reach the viable epidermis in porcine skin, contrary to the passively provided free or dendrimer complexed ASO, which was mainly localized to the stratum corneum. The iontophoretically delivered antisense oligonucleotide (ASO)–dendrimer complex reduced the tumor volume by 45%, consistent with the reduction in Bcl-2 protein levels and causing significant apoptosis in skin tumors [82]. Labala et al. developed layer-by-layer assembled gold nanoparticles (LbL-AuNP) to treat melanoma as a carrier for iontophoretic STAT3 siRNA delivery. AuNP coated with chitosan (AuNP-CS) were sequentially layered with siRNA and chitosan to form AuNP-CS/siRNA/CS. In vitro studies showed significant cell growth inhibition at 0.25 nM (49.0 ± 0.6%) and 0.5 nM (66.0 ± 0.2%) STAT3 siRNA concentration, respectively, and uptake of LbL-AuNP via clathrin-mediated endocytosis. LbL-AuNP reduced STAT3 protein expression and induced apoptosis in melanoma cells. Anodal iontophoresis further enhanced the skin penetration of LbL-AuNP to reach the viable epidermis. This approach can potentially treat melanoma through targeted siRNA delivery [83]**.**

The combined use of chemical penetration enhancers and iontophoresis has shown a significant increase in drug absorption through the skin compared to individual processes. Bhatia et al. investigated the effect of 5% terpenes (limonene, carvone, thymol, and cineole)/ethanol in combination with iontophoresis on LHRH permeation through the porcine epidermis. Terpenes, in combination with ethanol, enhanced LHRH flux compared to the control, with limonene showing the most significant improvement, a 4.7-fold increase compared to the control. Adding iontophoresis further improved LHRH flux, resulting in a 12.8-fold increase over the control [162]**.** Similarly, Smyth et al. studied the effect of chemical penetration enhancers and iontophoresis on LHRH permeation through the human epidermal membrane. Combining a 5% solution of oleic acid in propylene glycol (chemical penetration enhancer) with iontophoresis resulted in a 29.5-fold increase in permeability compared to passive permeability. In contrast, only iontophoresis showed a 14.3-fold increase and only the chemical penetration enhancer exhibited a 3.5-fold increase [85]. Liu et al. applied 0.1% Sodium lauryl sulphate, propylene glycol/70% ethanol (1:1), Isopropyl myristate/70% ethanol (1:1) as chemical penetration enhancers, slightly increasing drug penetration. Cathodal iontophoresis increased drug permeation threefold, while combining both approaches resulted in a four-fold enhancement in Gap junction protein connexin43 (Cx43) specific antisense oligodeoxynucleotide permeation. However, complete permeation across the skin was not achieved, and the molecules were either trapped in the epidermis or permeated deeply into the hair follicles (Fig. 3) [84].

### Ultrasound-assisted delivery

Ultrasound-assisted drug delivery typically utilizes lower frequencies, ranging from 20 kHz to 3 MHz, to enhance the skin's permeability and facilitate transdermal drug delivery. Ultrasound waves at these frequencies temporarily disrupt the skin barrier, allowing for better absorption of drugs through the skin [163]. The ultrasound waves induce the growth and oscillation of air pockets in the stratum corneum (SC), disrupting the lipid bilayer and enhancing skin permeability through a process known as cavitation. These changes occur superficially in the skin and get restored over time [164]. The frequency of ultrasound waves affects the extent of cavitation. Low-frequency, high-intensity ultrasound waves (2–50 W/cm^2^) create highly permeable localized transport regions (LTRs) in the stratum corneum. Besides cavitation, sonophoresis enhances transdermal delivery through thermal effects and radiation pressures [165]. Various researchers have studied ultrasound applications for the topical delivery of macromolecules. For instance, Tezel et al. investigated dermal oligonucleotide penetration using radiolabeled oligonucleotides in vitro. Ultrasound-assisted application for 10 min resulted in oligonucleotide concentrations ranging from ~ 0.5% to 5% of the donor concentration in superficial skin layers. Fluorescently labelled oligonucleotides formed non-uniform, localized transport pathways covering 5% of the exposed skin. Immuno-histochemical studies confirmed oligonucleotide penetration into these pathways without significant structural changes in the skin [87]. Park et al. conducted experiments with six Yorkshire pigs, splitting them into two groups. The control group (n = 3) received no ultrasound exposure with insulin, while the treated group (n = 3) received 20 kHz ultrasound with insulin at I(sptp) = 100 mW/cm^2^ and a 20% duty cycle for 60 min. An ultrasound transducer with insulin was applied to the pigs after anaesthesia. Blood glucose levels were monitored for 90 min, and the treated group exhibited a significant decrease in glucose levels over time [86]**.** Similarly in another study, Park et al. compared the physiological response of ultrasound-mediated transdermal insulin delivery to subcutaneously administered insulin in anaesthetized rats. The rats were divided into four groups, one receiving ultrasound-mediated insulin delivery and the others receiving subcutaneous insulin at different doses (0.15, 0.20, and 0.25 U/kg). Results showed minimal changes in blood glucose for lower subcutaneous doses, while ultrasound-mediated insulin delivery significantly reduced blood glucose levels, indicating a higher effective insulin dose was delivered [166].

Ultrasound-triggered macromolecule delivery system was developed by Di et al. which was capable of delivering insulin in a pulsatile manner. The encapsulated insulin in nanocapsules passively diffused from the nanoparticle but remained restricted within the microgel. The stored insulin in microgels is rapidly released upon ultrasound treatment to regulate blood glucose levels [88]. Liao et al. conducted a study to assess the effectiveness of a new wound healing treatment using US-mediated liquid-type epidermal growth factor (EGF)-coated lysozyme microbubble (LYMB) cavitation. The study revealed that the maximum loading efficacy of EGF onto LYMBs was 19.40 ± 0.04%. In vivo wound healing experiments showed a significant improvement in the recovery rate during the first 6 days (day 6: 54.28 ± 3.26%) compared to the control group (day 6: 26.36 ± 3.34%) when EGF-LYMB dressings were used with US exposure [89]. Hu et al. developed an ultrasound erosion protocol to generate a single-site, circular delivery region with a controlled size at the centre of patched skin. The study concluded that the application of shorter ultrasonic pulses (25 cycles) along with a higher pulse repetition frequency (4 kHz) and a more significant peak negative pressure (17.0 MPa) resulted in the formation of broader (0.995 mm) and deeper (300 mm) skin delivery zones, as well as a better success rate (94.44%). They were able to successfully deliver more than 1 µL of vaccine solution, which elicited immune responses against hepatitis B surface antigen successfully [167].

### Needle-free jet injection

The needle-free jet injection is a promising and non-invasive drug delivery method that offers numerous benefits in transdermal drug delivery. By eliminating the need for traditional needles, it reduces pain and anxiety for patients, making it especially advantageous for children and individuals with needle phobia. This technology allows for faster drug delivery and improved drug bioavailability through high-speed administration of drugs directly into the dermal or subcutaneous layer. While needle-free jet injection has certain limitations, such as skin irritation and bruising at the injection site, it is still beneficial in various medical applications, including vaccination, insulin delivery, and local anaesthesia administration. Continued research and development in jet injector design and formulation optimization hold promise for further enhancing its capabilities and widespread adoption in modern healthcare scenarios [91, 168]. Arora et al. proposed that pain and bruising caused by jet injections stem from deep skin penetration, which could be mitigated by reducing the penetration depth. They introduced a new approach, pulsed microjets, for drug delivery, enabling the precise entry of protein drugs into the skin without deep penetration. The microjets exhibited high velocity (v > 100 m/s), allowing access into the skin, but their small diameters (50–100 μm) and minimal volumes (2–15 nanoliters) restricted the penetration depth to approximately 200 μm. In vitro experiments confirmed the effective delivery of molecules into human skin, while in vivo experiments in rats demonstrated successful therapeutic insulin delivery [169]. Similar, phenomenon was used by Mooij et al. to delivered influenza vaccines intradermally into *Rhesus macaques* using pain- and needle-free jet injections. The injection protected against the influenza virus, with minimal and no increase in the IL6 and MCP-1 levels, respectively, compared to the control group treated with saline[90].

Multiple studies explored innovative delivery methods such as real-time controlled jet injection and jet injections combined with electroporation, showing enhanced immunization responses and improved therapeutic outcomes, including insulin delivery. For instance, Hogan et al. conducted a study to assess intradermal immunization against hepatitis B surface antigen (HBsAg) using a real-time controlled jet injector compared to intradermal and subcutaneous injection. Mice received three doses of aluminium-absorbed HBsAg at specific intervals. Only mice injected with the antigen exhibited detectable antibodies to HBsAg, with antibody levels increasing after subsequent injections. The immune responses of mice vaccinated via intradermal jet and subcutaneous needle injections were comparable at day 47, with only minor bleeding observed at the injection site [91]. In another study, Jiang et al. combined jet injections with electroporation for the delivery of DNA vaccine and observed that the combination multiplied the rate and magnitude of DNA vaccine-induced humoral and cellular responses compared to the jet injections alone, along with sustaining the antibody levels for over nine months [39]. Kong et al. observed that insulin delivery using jet injectors significantly lowered the 24 h mean glucose and maximum blood glucose in patients compared to the insulin pens. Moreover, there was no difference in the glycemic variability between the test and control groups and lower blood glucose in the jet injector group between 12 and 22 h [247]. A study by Hu et al. demonstrated that the needle-free injection of Human dermal fibroblasts exosomes resulted in an increased procollagen type I expression and a significant decrease in matrix metalloproteinase-1 (MMP-1) expression along with showing a higher level of dermal collagen deposition [92]. Chang et al. explored a needle-free pressure jet injection (PJI) system for DNA vaccine delivery. The study showed that PJI successfully delivered plasmid DNA to intradermal regions in rats and mice, resulting in higher protein expression than needle syringe injection. Animals receiving DNA vaccines via PJI exhibited dose-dependent production of anti-OVA antibodies, while needle syringe injection resulted in low antibody titers [170].

### Microneedles

**Fig. 5:**
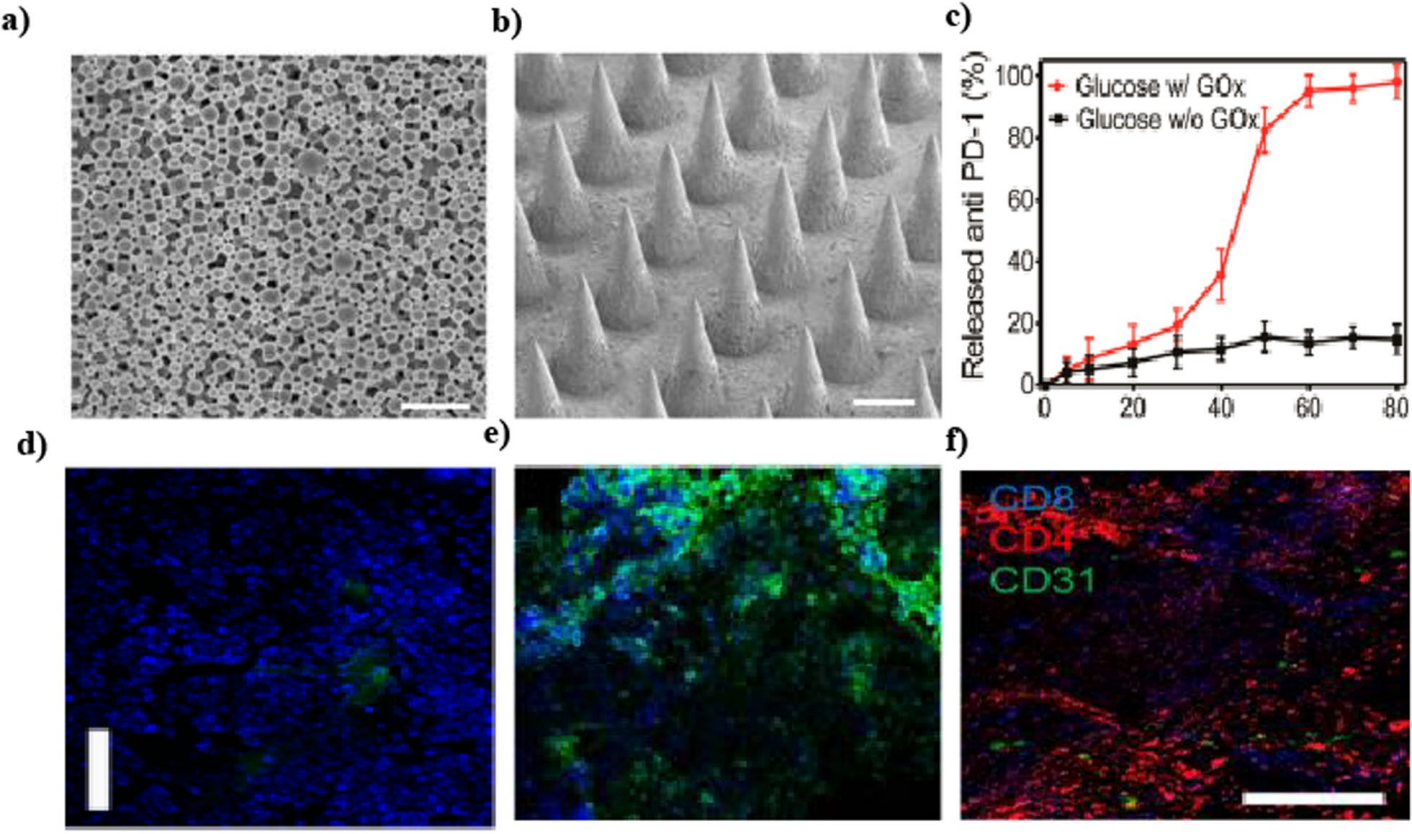
**a**, **b** SEM image of NPs and MNs; **c** In vitro accumulated aPD1 release from the MN patches incubated in 100 mg/dL glucose solution at 37 °C; **d**, **e** Immunofluorescence staining of tumors treated with free aPD1 and MN-GOx-aPD1 for 3 days; **f** Immunofluorescence of tumors showed CD4 + T cells and CD8 + T cells infiltration. Reprinted with permission from [180]

Microneedles are micron-sized structures that penetrate the stratum corneum, creating microchannels that enhance drug delivery, improve transdermal absorption, and facilitate vaccine administration. The aqueous pores afterwards help diffuse the drug from the patch into the deeper layers of the skin. The microneedles are 200–750 microns in length and consist of groups of 150–650 microneedles/cm^2^ called arrays, having a tip diameter of 25 mm, an interfacial area of 490 mm^2^, with an insertion force required of 0.058 N [171]. The approaches for the application of microneedles can be divided into five types: i) Poke with the patch approach—wherein the epidermal layer is excruciated with microneedles, and then the drug is applied in the form of a patch; (ii) Coat and poke approach—the drug is loaded into the needles, and the release of the same occurs by the dissolution of the microneedles. These provide an advantage of one-step application; however, the dose is limited due to the quantity that can be coated onto the total surface area of the microneedle projections; [172] (iii) Biodegradable Microneedle—the drug is loaded into the polymeric microneedles, which are biodegradable in nature, and (iv) Hollow Microneedle—the epidermis is punctured using the hollow microneedles, the drug is delivered via these holes [173] (v) Swellable microneedles—These are formed of suitable hydrogels poked inside the skin and swell up in situ. Water or interstitial fluid diffusion inside the microneedle array helps control the drug molecules through the system [174]. These can be utilized both as hypodermic needles and transdermal patches. The microneedles' morphology helps avoid contact with the nerve endings or cutaneous blood vessels, therefore helping in pain-free administration and avoiding the potential transmission of blood-borne infection [175]. For microneedles, potential risks include skin irritation, transient erythema, and the possibility of infection due to microchannel formation. However, these concerns can be mitigated with sterile manufacturing processes, appropriate material selection, and controlled application methods. Microneedles have shown significant potential in clinical applications, with ongoing trials and practical use cases highlighting their versatility [176]. For instance, microneedle-based patches are being tested for vaccine delivery, including influenza and COVID-19 vaccines, offering a minimally invasive alternative that enhances patient compliance [177]. Additionally, microneedles are being explored for transdermal insulin delivery in diabetes management, enabling controlled release and reducing the need for repeated injections. These clinical applications and trials demonstrate the translational potential of microneedles, bridging the gap between experimental research and real-world therapeutic use [178]. It is essential to maintain the required shape and size of the microneedles to overcome the skin's elasticity to enter the epidermis up to the required depth and remain intact for removal, unless in the case of dissolvable microneedles. It has also been observed that the pores caused by microneedles can close in around 2 h or up to 24 h in the case of occlusion [179]. Wang et al. developed biocompatible microneedles made of hyaluronic acid, incorporating pH-sensitive dextran nanoparticles (NPs) that encapsulated the anti-PD1 (aPD1) molecule. The microneedles painlessly penetrated the skin and efficiently delivered aPD1 to the tumor microenvironment, with the NPs in each needle containing both aPD1 and a glucose-specific enzyme for controlled and sustained release (Fig. 5) [180].

Bhatnagar et al. demonstrated that the application of zein microneedles (ZMNs) for transcutaneous vaccine delivery provided significantly (p < 0.001) greater antibody titers (total Immunoglobulin Gamma (IgG), Immunoglobulin Gamma-1 (IgG1), and Immunoglobulin Gamma-2α (IgG2α)) after the application of Ovalbumin-coated ZMNs and OVA intradermal injection compared with the control group [94]. In Shakya's study, the potential of microneedles for delivering peanut protein extract (PE) into the skin and inducing immune responses to desensitize peanut-sensitized mice was evaluated. The microneedle-treated mice exhibited significantly lower clinical symptoms of peanut-induced anaphylaxis, accompanied by down-regulation of systemic anaphylaxis mediators like histamine and mast cell protease-1 (MCPT-1) [95]**.** Liu et al. developed recombinant Staphylococcal enterotoxin B protein-loaded dissolving MNs made of chondroitin sulfate (2%) and trehalose (0.8%), which were capable of enhancing the in vivo retention time of the antigens, along with generating a high level of SEB specific antibody response which provided a 100% protection against a lethal SEB toxin challenge[96]. Yu et al. developed a single removable transdermal patch bearing microneedles loaded with insulin and a non-degradable glucose-responsive polymeric matrix, which was able to regulate the blood glucose levels in insulin-deficient diabetic mice and minipigs (with blood regulation lasting for > 20 h with patches of ~ 5 cm^2^) [249]. Qu et al. elaborated on the development of gelatin methacryloyl (GelMA) microneedle (MN)-based platform for local and controlled transdermal delivery of plasmid DNA (pDNA). The system exhibited high transfection efficiency in vitro and in vivo, producing a sustained release profile that could be modulated by adjusting the GelMA hydrogel's crosslinking degree [97]. Arikat et al. developed an intradermal Microneedle delivery device to target proinsulin and discovered that after a short insertion time (150 s), a considerable amount of the proinsulin (86%) was reproducibly delivered into local tissue. The coated MN system was used to deliver proinsulin to non-obese diabetic (NOD) mice, resulting in a considerable proliferation of adoptively transferred antigen-specific CD8 + T cells in the skin, demonstrating targeted delivery of the multi-epitope proinsulin antigen to skin resident APCs [248]. Boopathy et al. developed solid pyramidal microneedle (MN) arrays fabricated with silk fibroin protein tips encapsulating a stabilized HIV envelope trimer immunogen and adjuvant, supported on a dissolving polymer base, which was capable of implanting the vaccine-loaded silk tips upon brief skin application. Upon administration, the Env trimer was released over 2 wk in the skin, correlating with increased germinal centre (GC) B cell responses, a ∼1,300-fold increase in serum IgG titers, and a 16-fold increase in bone marrow (BM) plasma cells compared with bolus immunization [181].

Enhancing therapeutic penetration through the skin further led to the recent development of HA microneedles, presenting a minimally invasive and effective transdermal drug delivery approach. For instance, Liu et al. designed hyaluronic acid (HA)-based insulin-loaded microneedle arrays (MNs) for transdermal insulin delivery. In vivo studies on diabetic rats demonstrated rapid insulin release and self-dissolving properties of the MNs within 1 h, highlighting the synergistic effect of HA with insulin. The transdermal insulin pathway created by MNs disappeared after 24 h, indicating reversible skin damage. MN-administered insulin exhibited continuous hypoglycemic effects with lower peak plasma insulin levels but higher concentrations after 2 h compared to subcutaneous insulin administration at the same dose [182]. In a study conducted by Mönkäre et al., dissolving microneedles (MNs) based on hyaluronan (HA) were developed, loaded with PLGA nanoparticles co-encapsulating ovalbumin (OVA) and poly(I: C) for intradermal immunization. The penetration efficiency of these dissolving MNs into ex vivo human skin was evaluated using a trypan blue assay. MNs with NP: HA weight ratios of 1:1, 1:4, and 1:10 demonstrated skin penetration efficiencies of 91.9 ± 1.6%, 100%, and 85.4 ± 2.5%, respectively. The MNs with an NP: HA ratio of 1:4 exhibited excellent skin penetration efficiency [183]. Naito et al. developed self-dissolving microneedle arrays using Hyaluronic acid to enhance transdermal absorption of human parathyroid hormone (PTH). The microneedles provided stability due to their dissolvable nature. The bioavailability of PTH, relative to subcutaneous injection, was 100%, and the PTH-loaded MNs effectively preserved bone density in a rat osteoporosis model without causing skin irritation. These findings highlight the potential of transdermal PTH delivery in osteoporosis treatment [184]. Chiu et al. developed a unique composite microneedle (MN) formulation, incorporating a sodium hyaluronate (HA) tip and a chitosan base, which enabled biphasic antigen release for intradermal single-dose vaccination. The dissolvable HA tip facilitated the rapid release of encapsulated antigens, triggering an immune response, while the biodegradable chitosan base provided sustained antigen release for 4 weeks, further enhancing immunity. The HA/chitosan MN, containing ovalbumin (OVA), elicited robust and long-lasting T helper type 1 (Th1) and Th2 immune responses in rats, surpassing traditional subcutaneous vaccinations in terms of antibody responses. This highlights the significant role of hyaluronic acid in promoting skin permeation and improving vaccination efficacy [185]. Lee et al. conducted a study to develop a dissolving microneedle (MN) patch containing Glutathione (GSH) and hyaluronic acid (HA), aiming to enhance skin permeation and reduce the unpleasant odour of GSH. HA was chosen to create odourless GSH solutions and serve as a carrier for GSH in the MN fabrication process. The GSH-loaded MN arrays, with up to 10% GSH, exhibited uniform patterns and suitable mechanical properties for skin insertion. These GSH-MNs dissolved within 10 min upon insertion into porcine skin, releasing GSH without oxidation, showcasing the significance of hyaluronic acid in facilitating efficient skin penetration [186].

### Laser-assisted delivery

Laser-assisted delivery involves delivering macromolecules across the skin using erbium: yttrium-gallium garnet (Er: YAG, 2940 nm), yttrium-scandium-gallium-garnet (YSGG, 2790 nm), and CO_2_ (10,600 nm) lasers. Laser-assisted delivery employs three mechanisms: direct ablation, photomechanical wave (PW)-induced breakdown, and the photothermal effect. In direct ablation, the laser decomposes the skin target into small fragments, moving away from the skin surface at supersonic speed [187] This reduces the skin barrier function, enabling efficient transport of macromolecules. A photomechanical wave is a broadband, unipolar, compressive wave generated by lasers. It temporarily permeabilizes the cell membrane and skin surface but cannot peel the SC. Lipid disruption in the SC and expansion of lacunar space help form transient pores for drug delivery [188]. Photomechanical waves also affect the cell plasma membrane, opening transcellular routes for drug transport [189]. Photothermal is a property of lasers like CO_2_ laser, where absorbed light energy converts to heat, heating the skin tissue and causing vaporization if delivered in high quantity quickly [190]. Several studies explored lasers to enhance macromolecule delivery across the skin. Fang et al. showed Er:YAG laser enhanced hexameric insulin delivery across the skin without altering viable skin morphology and facilitated transdermal delivery of FITC-labelled dextran (molecular weight ≥ 77 kDa)[99]. Lee et al. investigated Er:YAG laser's potential for transdermal drug delivery. They found a 3–30-fold increase in antisense oligonucleotides permeation, DNA expression from epidermis to subcutis, and 3–140 fold enhancement in peptide permeation [191]. Lee et al. compared Er:YAG and CO_2_ lasers for siRNA penetration. Er:YAG at 5 J/cm^2^ and CO_2_ at 4 mJ/400 spots showed 11-fold and 12-fold improvements in siRNA flux, respectively, compared to untreated skin. Successful penetration of siRNA into the skin with fractional lasers opened new possibilities for skin-related therapeutic interventions [98]. Bachhav et al. evaluated the effect of Er: YAG diode-pumped laser application on delivering recombinant human growth hormone (hGH; 22 kDa), urinary follicle-stimulating hormone (FSH; 30 kDa), and FITC-labelled bovine serum albumin across the skin. Laser application increased permeation to 8.1 ± 4.2, 0.2 ± 0.1, and 273.3 ± 30.6 µg/cm^2^ for hGH, FSH, and FITC-BSA, respectively, compared to no permeation in the control group [100]. Yu et al. studied the effect of fractional laser ablation on Antithymocyte globulin (ATG) and Basiliximab delivery, increasing total antibody delivery for ATG from 1.18 ± 0.10 to 3.98 ± 0.64 and 4.97 ± 0.83 μg/cm^2^, respectively. These corresponded to a 19.7, 66.3, and 82.8-fold increase over the control (untreated skin) [192]. Fujimoto et al. concluded that Fractional laser irradiation increased the skin permeation rate of fluorescein isothiocyanate-conjugated ovalbumin OVA-FITC liposomes (4–40%) in a dose-dependent manner. At higher laser energy levels, drug permeation (7–8%) was similar in peeled or untreated skin compared to enhancement due to stratum corneum peeling at lower energy levels. Increasing laser power and irradiation time improved liposome uptake by cells and peptide drug penetration across the skin in a dose-dependent manner. High-energy CO_2_ fractional laser effectively overcame the rate-limiting barrier function of the stratum corneum [193].

### Electroporation

Electroporation employs high-voltage electric pulses (typically ranging from 100 to 1000 V) of short duration (microseconds to milliseconds) to temporarily permeabilize cell membranes or the skin. This process creates transient pores and enhances permeability, facilitating the transport of large molecules such as drugs or genes into deeper layers of the skin. Electroporation efficiency is influenced by electrical parameters (e.g., field strength, pulse duration, pulse number) and the physicochemical properties of the delivered molecule. Initially developed for cell transfection, electroporation has evolved into a versatile and efficient method to enhance stratum corneum permeability. It achieves this by widening existing channels and forming new pathways in the stratum corneum, enabling molecular transport through mechanisms such as electrophoretic mobility, increased diffusion, and electroosmosis [194]. Electroporation has been successfully applied beyond insulin delivery, facilitating gene electrotransfer (e.g., IL-12 plasmids) and enhancing the transdermal administration of chemotherapeutics such as Bleomycin and Cisplatin. It also plays a crucial role in electrochemotherapy (ECT), an established treatment for tumors and superficial metastases in both veterinary and clinical settings [194]. Electroporation is generally well-tolerated, with side effects such as erythema and discomfort mitigated by using non-invasive electrodes, optimized electroporation parameters, and mild local anesthesia. Clinical studies report high patient satisfaction, with 87% of patients expressing willingness to undergo ECT again, if clinically indicated. ECT has shown efficacy in treating various cancers, including metastatic breast cancer, soft tissue sarcoma, squamous cell carcinoma, basal cell carcinoma, and malignant melanoma. These findings underscore the versatility, safety, and therapeutic potential of electroporation for a wide range of medical applications, particularly in cancer treatments and transdermal delivery systems. [195]. For electroporation, safety concerns such as erythema, mild pain, and muscle contractions are noted. These effects can be minimized by optimizing electrical parameters, using non-invasive electrodes, and employing local anesthesia where necessary. Electroporation can improve the transdermal delivery of molecules with a size of at least 40 kDa, although permeability reduces with larger molecular sizes [163]. For instance, Riviere et al. observed nearly a four-fold increase in maximal LHRH concentration in the perfusate following electroporation, showing rapid onset within 10 min after the pulse [196]. In another study, Sen et al. found that FITC-insulin retained in the epidermis after electroporation was primarily transported through lipid multilayers around the corneocytes [197].

Combining iontophoresis and electroporation, Chang et al. enhanced the transdermal delivery of salmon calcitonin and parathyroid hormone (1–34) compared to either method alone [198]. Zhao et al. developed a needle-free transcutaneous electroporation method for delivering peptide vaccines using SIINFEKL, inducing an equivalent peptide-specific CTL response [199]. Mohammad et al. observed that insulin delivery with electroporation significantly decreased blood sugar levels compared to control groups, and the best performance was with insulin solution combined with electroporation in various conditions [101]. Electroporation can potentially improve the transdermal delivery of proteins and peptides, offering controlled and pulsatile drug delivery. However, further exploration is needed to ensure the safety of this technique for clinical applications due to potential side effects such as erythema, muscle contractions, and pain.

### Combination therapy

Researchers have explored innovative combinations of mechanical techniques to enhance the transdermal delivery of macromolecules. Zheng et al. developed a wearable iontophoresis-driven microneedle (MN) patch for active and efficient transdermal delivery of vaccine macromolecules. This system synergistically combined microneedles to breach the stratum corneum and iontophoresis to drive vaccine molecules through the skin actively. The study demonstrated that the amount of ovalbumin delivered could be precisely controlled by adjusting the iontophoresis current. In vivo immunization studies in BALB/c mice revealed that transdermal vaccination using this system induced a stronger immune response than traditional intramuscular injections [200]. Jiang et al. investigated the impact of combining needle-free jet injection with electroporation (Jet-EP) for DNA vaccine delivery. Their study demonstrated that the addition of electroporation significantly enhanced in vivo DNA transfection efficiency in rabbit muscle compared to jet injection alone. Jet-EP delivery further augmented the rate and magnitude of humoral and cellular immune responses in both rabbits and nonhuman primates (NHPs), leading to higher proportions of polyfunctional antigen-specific T cells producing IFNγ, IL-2, and TNFα. Notably, NHPs immunized with a DNA vaccine using Jet-EP delivery sustained elevated antibody levels for nine months, far outperforming jet injection [39]. The study by Amano et al. demonstrated that MN application efectively disrupted the skin barrier function. This pretreatment signifcantly enhanced iontophoretic pilocarpine-induced palmar sweating. Interestingly, similar MN pretreatment on forearm skin did not afect pilocarpine-induced sweating. Notably, the observed palmar sweating increased without a corresponding increase in pilocarpine delivery. These findings suggest that MN pretreatment enhances sweat responses in palmar skin, potentially via mechanisms beyond increased drug penetration. IP/EP-assisted MN approaches have shown significant promise in enhancing TDD for various skin conditions [201].

## Nanosystems

Nanotechnology presents a promising field with vast potential for drug delivery, offering innovative solutions to conventional limitations. In topical drug delivery, nanoscale materials like nanoemulsions, solid lipid nanoparticles, niosomes, ethosomes, transferosomes, liposomes, etc., have gained attention for enhancing macromolecule permeation through the skin [202]. Researchers have harnessed these nanosystems to improve drug delivery efficacy and precision. Targeted and controlled release of macromolecules becomes achievable, overcoming stability, bioavailability, and penetration depth challenges. However, maintaining the integrity of encapsulated macromolecules poses challenges, including degradation from environmental factors, carrier-material compatibility, aggregation, and premature release. Nanomaterials' tunable characteristics enable delicate macromolecule encapsulation and protection during transit, preserving their integrity and therapeutic activity [197]. Nanotechnology's versatility allows tailored drug release profiles, sustained release, and site-specific targeting, transforming topical macromolecular delivery [203].

### Nanoemulsion

Nanoemulsions consist of submicron-sized oil and water particles stabilized by surfactants. These are formed using high or low-energy emulsification techniques and usually contain a low surfactant percentage [204]. The small droplet size ensures stability, resisting destabilization due to gravitational separation, flocculation, and coalescence. The low surfactant concentration provides an additional advantage for topical delivery, reducing skin irritancy [205]. Nanoemulsions enhance drug permeation into the skin through various mechanisms. Further, large surface area, good skin contact, and occlusive nature ensure adequate contact with the stratum corneum surface. Additionally, oily and surfactant components enhance drug permeation [206]. NEs has been explored for topical delivery of proteins. For instance, plasmid expressing chloramphenicol acetyltransferase (CAT) or human interferon-alpha2 (IFN-2) cDNA was produced as water-in-oil nanoemulsions and applied to the skin of mice. This resulted in ideal 24-h skin transgenic expression and a significant build-up of human interferon-2, with no apparent toxicity or irritation associated with short-term application [67]. Further, Tamayo et al. present a nanoemulsion composition for topical vaccine administration, utilizing outer membrane vesicles. The application of this nanoemulsion, containing *Salmonella enterica* outer membrane antigens (size 20–100 nm) induced a specific antibody response. Compared to other formulations, the nanoemulsion's occlusive effect, along with Labrasol® and Plurol® oleique as penetration enhancers, facilitated antigen uptake through the epidermal and transfollicular routes. However, when poly (anhydride) nanoparticles loaded with antigens were incorporated into the nanoemulsion, the specific IgG response in serum decreased due to their larger size [103]. In another study, Choi et al. focused on enhancing macromolecular topical delivery through a nanoemulsion (NE) system loaded with LMWP-conjugated growth factors for wound healing. Synergistic effects were found with a 100:100:10 concentration ratio of LMWP-EGF, LMWP-IGF-I, and LMWP-PDGF-A. The NE-loaded hydrogel, with an average droplet diameter of 127 ± 4.30 nm demonstrated significantly higher skin permeation of LMWP-conjugated growth factors than native ones [102].

### Nanoparticles

**Fig. 6:**
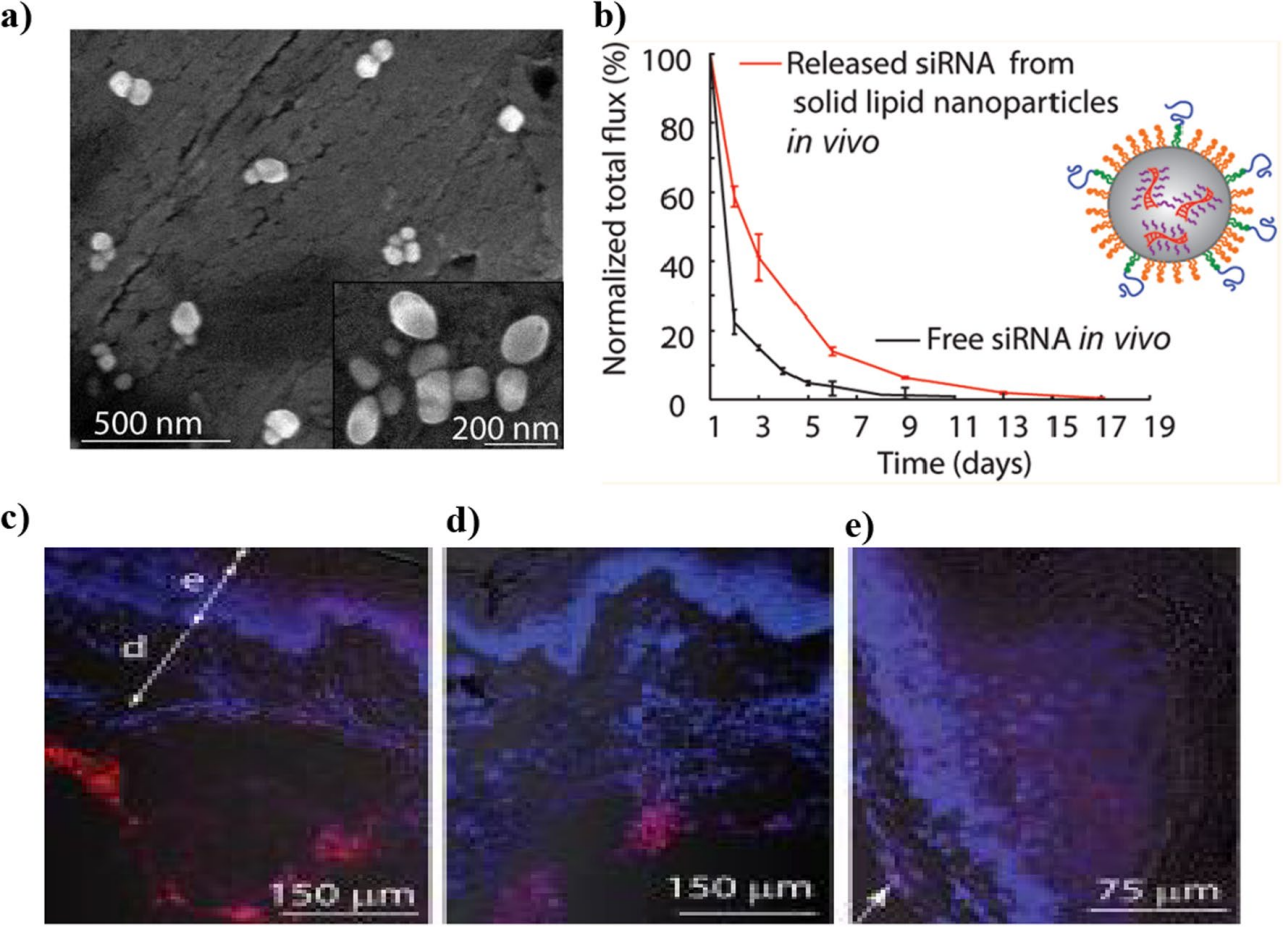
**a**, **b** SEM image and In vivo siRNA release; **c**-**e** Fluorescence microscopy of skin sections prepared from mice treated with siGLO red nanoparticles. Reprinted with permission from [104]

Solid lipid nanoparticles are composed of lipids, lipid-like materials or a mixture of the same, having a diameter between 50 and 1000 nm. These comprise a solid core matrix stabilized by surfactants that solubilizlipophilic molecules [102]. A broad array of lipids is utilized in crafting Solid Lipid Nanoparticles (SLNs). These encompass diverse triglycerides (e.g., tristearin), diglycerides (e.g., glycerol behenate), monoglycerides (e.g., glycerol monostearate), fatty acids (e.g., stearic acid), steroids (e.g., cholesterol), and waxes (e.g., cetyl palmitate). This wide lipid selection grants adaptability in tailoring SLN characteristics for varied applications. Tristearin, for example, fosters stability; cholesterol enhances membrane permeability, while cetyl palmitate facilitates sustained drug release [207]. Solid Lipid Nanoparticles (SLNs) have been extensively investigated for the delivery of macromolecules such as oligonucleotides, proteins, and peptides, owing to their ability to enhance stability, improve bioavailability, and provide controlled release. For instance, Lobovkina et al. showcased successful siRNA integration and long-term release via SLNs. Nanoprecipitation was employed to create Tristearin-based SLNs, efficiently accommodating siRNA (loading ratio of 4.4–5.5 wt %) through a hydrophobic ion pairing method with the cationic lipid DOTAP. Injections of these nanocarriers into mouse footpads resulted in extended siRNA release for 10–13 days [104]^.^Hwang et al. conducted permeation studies to assess the skin accumulation of recombinant human epidermal growth factor (rhEGF) using solid lipid nanoparticles (SLNs). The recovery (%) of rhEGF from diluted rat skin homogenates varied from 99% −115.27%, exhibiting an accuracy range of around 80% −120%. Notably, there was no detectable rhEGF permeation through rat skin tissue. Nonetheless, the amount of rhEGF accumulated within rat skin significantly increased when loaded into SLNs compared to rhEGF mixtures with SLNs vehicle or free rhEGF solution [246]. Another study by Suter et al. discovered that incorporating amorphous heptapeptide DEETGEF into the SLNs induced the activation of NQO1 (NADPH quinone oxidoreductase), HMOX1 (Heme oxygenase-1), and PRDX1 (Peroxiredoxin-1) genes, which function as cell-protecting enzymes. In an ex vivo study with skin explants, they successfully stimulated NQO1, HMOX1, and PRDX1 genes, all cell-protecting enzymes [106] (Fig. 6).

Apart from SLNs, polymeric nanoparticles have been explored that provides unique benefits in encapsulating and releasing diverse therapeutic agents. Polymeric nanoparticles offer controlled particle size, surface properties, and release of pharmacologically active agents, enabling targeted drug action at the desired therapeutic rate, improving drug/protein stability and allowing controlled release [208]. For instance, Cui et al. developed chitosan-based nanoparticles with plasmid DNA (pDNA) to explore genetic immunization. They found that these nanoparticles effectively stabilized the pDNA in serum. Moreover, when topically applied, the nanoparticles led to detectable and quantifiable luciferase expression levels in mouse skin within 24 h. Additionally, they triggered a significant antigen-specific IgG titer for expressed β-galactosidase at 28 days [64]. Mattheolabakis et al. noted that OVA-loaded PLA nanoparticles were found in the hair follicle ducts after being applied to the exposed skin of mice in vivo. This demonstrates the nanoparticles' capability to penetrate the skin barrier through hair follicles. Additionally, when delivered through an alternate route, these nanoparticles could trigger a strong cellular response upon administration and effectively prime against a challenging dose of Ovalbumin [105]. Mittal et al. conducted a study that found the protection of ovalbumin through encapsulation in PLGA nanoparticles and chitosan PLGA nanoparticles. The encapsulation retained its biological activity at 74% (PLGA) and 64% (Chit-PLGA), preventing cleavage and aggregation. Additionally, the study revealed an enhanced delivery efficiency of Ovalbumin into hair follicles on excised pig ears, with a 2–threefold improvement compared to the OVA [209]. Luo et al. formulated ε-polylysine-g-cetyl nanoparticles, showcasing impressive insulin loading ability (up to 17%). These nanoparticles penetrated the stratum corneum, delivering active insulin to the dermis without compromising the epidermis integrity. This pioneering method holds great potential for efficient transdermal insulin delivery, promising significant benefits in diabetes management [210]

### Liposomes

Liposomes, composed of phospholipids and cholesterol from natural or artificial sources, are biocompatible carriers capable of transporting diverse drug payloads. Their unique composition enables efficient encapsulation and targeted delivery of therapeutic agents while offering protection against degradation, controlled release, and enhanced therapeutic efficacy. This versatility makes liposomes a highly effective and widely adopted drug delivery system [48]. Thanks to their versatility, liposomes make excellent drug delivery systems, offering protection, controlled release, and targeted administration of therapeutic agents [211]. For example, Li et al. showed that entrapping pigment melanin into phosphatidylcholine liposomes improved molecule delivery to mouse hair follicles and shafts upon topical application. Negligible amounts of delivered molecules in the dermis, epidermis, or bloodstream further confirmed follicular enrichment. Charged based liposomes are also employed for the macromolecular delivery [212]. In a study, Song et al. formulated cationic, neutral, and anionic flexible liposomes to improve the topical delivery of low-molecular-weight heparin (LMWH). They found that cationic liposomes had a three-fold higher entrapment efficiency of LMWH and superior physicochemical stability compared to the other variants. Moreover, the cationic liposomes enhanced in vitro skin penetration and in vivo localization into deeper skin layers [107]. Considering the same molecule, Song et al. compared the low molecular weight heparin (LMWH)-loaded flexible liposomes (flexosomes) with ethosomes and observed that the flexosomes demonstrated a 2.6-fold higher permeability coefficient than ethosomes along with a 3.2-fold increase in the skin deposition of flexosome as compared to an only 2.0-fold increase by ethosomes. The AUC_0–24 h_ was also 2.5-fold higher than the ethosomes, indicating the superiority of flexosomes compared to the ethosomes [213].

**Fig. 7:**
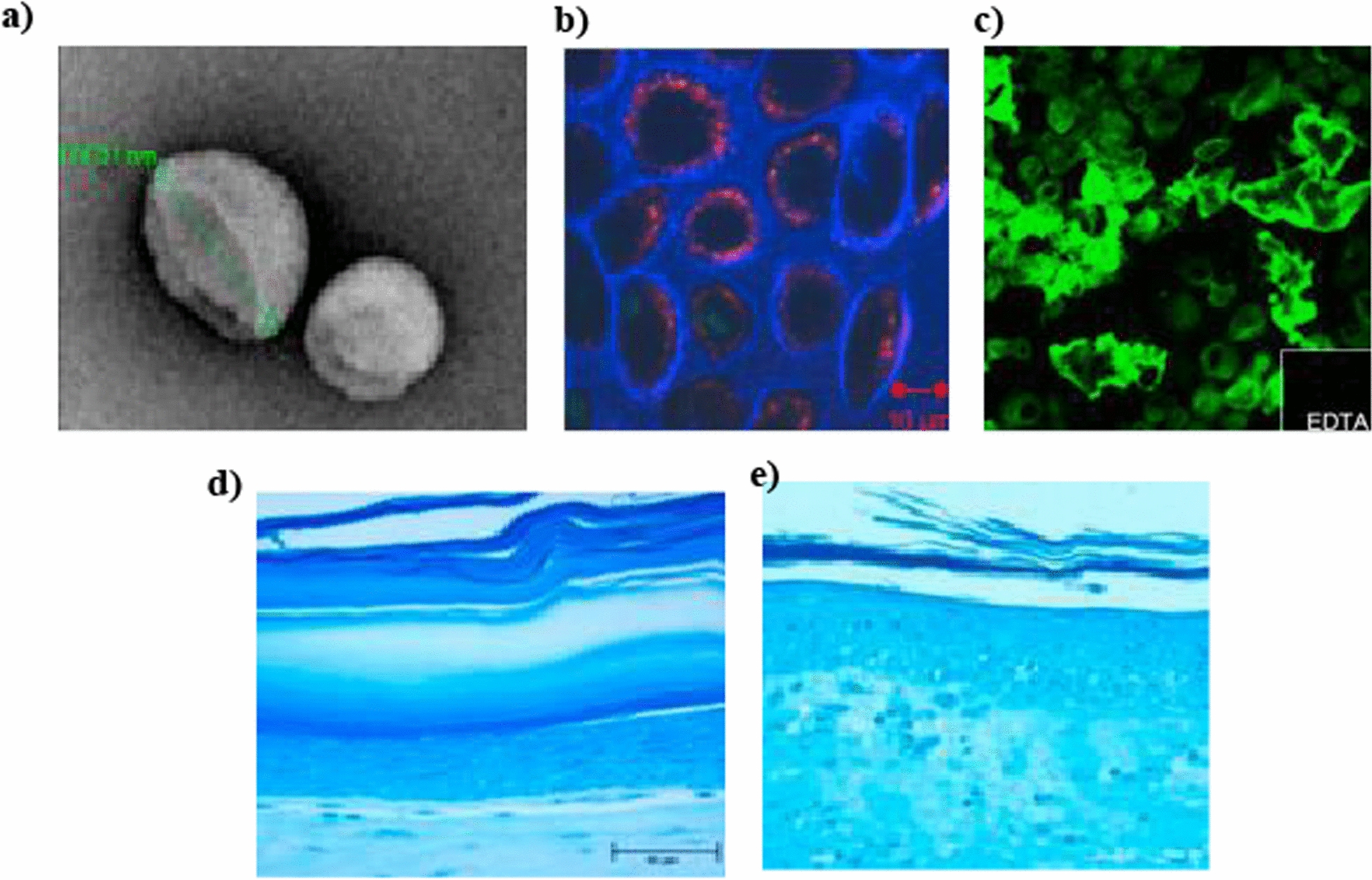
**a** SEM image of Vector-Coupled Liposome; **b** Internalization of rhTG1 LUVs into primary keratinocytes was measured via CLSM; **c** Intracellular Activity of rhTG1 after Internalization into Primary Keratinocytes; **d**, **e** Histology of Regenerated TG1-Deficient Skin Grafts in a Skin-Humanized Mouse Model before and after Application of rhTG1 LUV. Reprinted with permission from [108]

Li et al. observed that administering Ovalbumin enclosed in flexible liposomes via transcutaneous means yielded a potent immune response comparable to subcutaneous OVA injection with Al(OH)_3_ adjuvant. Furthermore, co-administering imiquimod with the Ovalbumin-loaded liposomes enhanced transcutaneous immune responses. The study also detected fluorescence-labelled liposomes in hair follicle ducts in vivo, demonstrating the flexible liposomes' ability to breach the skin barrier through hair follicles [250]. In another study, Aufenvenne et al. devised sterically stabilized liposomes containing recombinant human TG1 (rhTG1) for treating Transglutaminase-1 (TG1)-deficient autosomal-recessive congenital ichthyosis (ARCI). These liposomes effectively conveyed rhTG1 to primary keratinocytes, restoring TG1 activity and epidermal barrier function. In a skin-humanized mouse model, the application of rhTG1 liposomes significantly ameliorated the ichthyosis phenotype, normalized the ARCI skin, and eliminated cholesterol clefts ultrastructurally [108]. Desmet et al. successfully demonstrated targeted delivery of RNAi molecules to the epidermis of both damaged and intact human skin using 'DDC642', a liposomal carrier. However, penetration into the dermis or circulatory system was not achieved. As a proof of concept, DDC642-delivered siRNA effectively down-regulated the psoriasis marker human beta-defensin 2 in a psoriasis tissue model. In a Franz diffusion cell set-up, no detectable Cy5 signal was observed in the receptor compartments after 6 h of treatment on normal human skin (intact or tape-stripped), indicating minimal or no penetration (< 50 pm) of the siRNA-containing lipoplexes into the dermis [109]. Dorrani et al. accomplished the development of liposome-siRNA complexes that can internalize into melanoma cells. These complexes effectively suppressed BRAF protein expression and promoted cell death in melanoma cells. This showcased the formulation's capacity to transport siRNA across the stratum corneum and deposit it at the lower epidermis/upper dermis. By conducting skin permeation studies, the researchers found that liposomes containing an 8:1 ratio of DOTAP to NaChol and siRNA ratios of 8:1, 12:1, and 16:1 demonstrated the highest rates of permeation and deposition in the upper dermis. These findings highlight the formulation's potential for successful topical siRNA delivery [214] (Fig. 7).

### Niosomes

Niosomes, similar to liposomes, consist of nonionic surfactants and cholesterol, forming nanosized vesicles with unique structural components. The bilayer membrane, composed of amphiphilic nonionic surfactants and cholesterol, encloses a hydrophilic core suitable for encapsulating water-soluble macromolecules, while the hydrophobic region accommodates lipophilic macromolecules. In the realm of topical macromolecule delivery, niosomes offer remarkable advantages. Their nonionic nature reduces interactions with charged macromolecules, enhancing stability and minimizing degradation risks [215]. Furthermore, niosomes protect delicate macromolecules such as proteins, peptides, and nucleic acids, improving their bioavailability and skin penetration due to their nanoscale size and ability to fuse with the stratum corneum [216]. Customizing niosomes with optimized surfactant-cholesterol ratios makes specific tailoring for diverse delivery requirements possible, rendering them promising and effective drug delivery carriers for macromolecules in pharmaceutical and biomedical research [217]. For instance, Vyas et al. demonstrated that DNA encoding hepatitis B surface antigen (HBsAg) was encapsulated in niosomes, and showed that this formulation could induce a serum antibody titer and endogenous cytokine levels comparable to intramuscular recombinant HBsAg and topical liposomes [70]. Further, Maheshwari et al. investigated topical vaccine delivery utilizing niosomes, incorporating hepatitis B surface protein as an antigen and cholera toxin B as an adjuvant. The optimized niosomal formulation successfully entrapped 58.11 ± 0.71% of the antigen, featuring vesicles within the 2.83 ± 0.29 μm range. In vitro permeation and skin deposition analyses on human cadaver skin demonstrated effective skin penetration by the niosomes. The outcomes indicated that topical administration of the niosomes significantly enhanced serum IgG titers compared to single administration, thereby effectively stimulating serum immune responses. Furthermore, the higher IgG1/IgG2a ratio implied that cholera toxin B mixed niosomes elicited Th1 and Th2 responses, suggesting their potential as an efficient topical vaccine delivery system [218]. In another study, Manosroi et al. synthesized elastic anionic niosomes using cholesterol, and dicetyl phosphate (molar ratio 1:1:0.05 at 20 mM). These niosomes incorporated varying concentrations of ethanol and edge activators (sodium cholate and sodium deoxycholate) and exhibited larger vesicular size (ranging from 171.94 ± 63.52 to 683.17 ± 331.47 nm) and higher negative zeta potential (from − 6.45 ± 2.76 to − 17.40 ± 2.51 mV) compared to nonelastic niosomes. Elastic niosomes, except for NaDC vesicles, displayed superior elasticity and entrapment efficiency. When used for topical delivery, Tat–green fluorescent protein-loaded elastic niosomes (containing 1 mol% NaC) demonstrated the highest cell viability (HT-29: 92.32 ± 3.82%, KB cells: 96.62 ± 5.96%) and transdermal flux (10.46 ± 3.45 μg/cm^2^/h) in rat skin, suggesting their potential for effectively delivering therapeutic macromolecules [219].

Shinde et al. observed that the encapsulation of proteolytic enzyme serratiopeptidase in niosomal gel resulted in a four-fold increase in the steady-state flux of serratiopeptidase, along with a significant increase in the permeation of the same. Moreover, the in vivo efficacy studies indicated the presence of a topical anti-inflammatory activity similar to that of diclofenac gel[110]. Shehata et al. explored the effects of various formulation parameters on the in vitro release, viscosity, and penetration of an insulin niosome emulgel. The permeation sequence observed was as follows: insulin niosome emulgel > insulin niosome gel > insulin emulgel > insulin gel > insulin niosomes > insulin solution. Notably, the insulin niosome emulgel exhibited a tenfold increase in transdermal insulin flow compared to the control (insulin solution). In contrast, niosomes alone showed a modest 1.45-fold enhancement in insulin permeability. The steady-state transdermal flux (SSTF) of insulin from the niosome preparation was 13.17 µg/cm^2^/h, and only 9 µg/cm^2^/h from the insulin solution. This significant improvement in permeation by niosomes may be attributed to their potential to extract skin lipids or disrupt corneocytes, thereby facilitating insulin penetration through the skin [220].

### Ethosomes

Ethosomes, advanced lipid-based nanocarriers, enable effective topical delivery of macromolecules. Incorporating high ethanol concentrations with phospholipids enhances lipid fluidity and drug encapsulation efficiency. Ethosomes surpass traditional liposomes in skin penetration, facilitating the transport of macromolecules (e.g., proteins, peptides, nucleic acids) through the stratum corneum to deeper skin layers [221]. Moreover, sustained drug release leads to increased bioavailability and prolonged therapeutic effects. These features make ethosomes an attractive, non-invasive approach for topical drug delivery, reducing the need for invasive administration methods. Additionally, their smaller and more stable nature enhances skin penetration and drug delivery properties [222]. For instance, Chen et al. developed a DOTAP-based ethosomal carrier system containing siRNAs, leading to a 6.3 ± 1.7-fold increase in siRNA penetration into porcine skin in vitro. Additionally, it showed a ten-fold rise in epidermis siRNA accumulation compared to the aqueous solution. Incorporating SPACE peptides resulted in an 83.3 ± 3.0% knockdown relative to the control and in vivo experiments with female BALB/C mice confirmed the efficacy of the siRNA delivery system [71]. In another study, Kim et al. prepared transformer-ethosomes which were observed to enhance the skin permeation of palmitoyl pentapeptide through either an artificial membrane or human cadaver skin to a much higher level than the palmitoyl pentapeptide solution and liposomes [111]. A study by Fu et al. indicated that the loading of Thymosin β−4 into the ethosomal gels was able to enhance the cumulative release of the protein by 1.67 fold than that of the T-β4 gel in vitro release study in 5 h, along with being able to reduce the wound healing time by half as observed in the in vivo study [112]. Yang et al. investigated transcutaneous immunization (TCI) utilizing modified Eth-HA-GC/SF mats to surmount the skin's barrier, the stratum corneum (SC), for optimal antigen delivery. The Eth-HA-GC compound exhibited favourable stability and heightened dendritic cell (DCs) uptake. OVA@Eth-HA-GC/SF mats demonstrated proficient transdermal performance, triggering immune responses, and fostering serum anti-OVA-specific IgG production and cytokine expression. Moreover, in a murine model, the mats effectively restrained tumor growth. Overall, the study underscores the promising potential of this system for improved permeation strategies [223].

### Transferosomes

Transferosomes, specialized lipid-based vesicles for transdermal drug delivery, comprise phospholipids and edge activators, like surfactants or bile salts, to enhance macromolecule penetration through the skin. Their flexibility allows them to traverse the lipid layers, surmounting the stratum corneum barrier and delivering therapeutics effectively [224]. Transferosomes offer advantages like improved skin permeation, extended drug release, and reduced systemic side effects in the topical delivery of macromolecules. They ensure non-invasive administration and better patient compliance, encapsulating diverse hydrophilic and hydrophobic drugs, thereby enhancing versatility. Nevertheless, complex formulation demands precise optimization for specific drugs, and stability issues and drug leakage may affect shelf life [225, 226]. For instance, Gupta et al. investigated transfersomes to deliver the antigen tetanus toxoid (TT) topically to the skin. These transfersomes effectively penetrated intact skin, reaching immunocompetent Langerhans cells and providing the antigen. Topical administration of TT-loaded transfersomes elicited a robust immune response, producing serum anti-tetanus toxoid IgG [227]. In another study, Malakar et al. discovered that loading insulin in tranfersomes resulted in a permeation flux of 13.50 g/cm^2^/h, a drug entrapment efficiency of 56.55%, and a zero-order pattern of release. Furthermore, iontophoresis increased the penetration flow to 17.60 g/cm^2^/h, with the in vivo investigation revealing a persistent hypoglycemia impact in diabetic rats, 24 h after transdermal delivery [228]. In another study, Marwah et al. prepared insulin-loaded transferosome gel with 78% entrapment efficiency and a cumulative percent drug release of 83.11%. The study further elaborated that the loading of insulin into the tranferosomes was able to produce a better glucose-lowering effect as compared to the control gel [229]. Shamshiri et al. prepared growth hormone-loaded tranfersome formulations that enhanced the transport of human growth hormone (hGH) in the rat skin as compared to the hGH alone. Additionally, the incorporation of distinct surfactants (F1_sodium deoxycholate and F2_sodium lauryl sulfate) resulted in transdermal delivery rates of 489.5 and 248.46 ng cm^−2^, respectively [230]. In another study conducted by Surini et al., it was found that loading recombinant human epidermal growth factor (rhEGF) into transferosomes significantly boosted skin penetration by 5.56 times compared to non-transpersonal emulgel. The study also revealed that the rhEGF levels remained within the range of 84.96%–105.73% and 54.45%–66.13% after 3 months of storage at 2 °C–8 °C and 25 °C/RH 60%, respectively. These results suggest that transferosomes improve skin penetration and offer satisfactory stability, making them a promising option for advanced topical drug delivery applications [231].

### Biphasic vesicles

Biphasic vesicles are distinctive structures that blend liposomes and emulsions, with a size range of 1–100 µm, dependent on the utilized materials and drugs. These vesicles encompass aqueous, oily, or micellar segments encircled by concentric phospholipid bilayers. Research has identified multilamellar lipid bilayer scattering (Bragg reflections) and a wide scattering peak from submicron emulsion droplets within these systems [232]. Notably, the overall scattering pattern of biphasic vesicles deviates from that of a mixture of preformed liposomes and emulsion, signifying the effective solubilization of submicron emulsion droplets within the biphasic vesicles. Typically, these vesicles consist of a phospholipid phase (including soya phosphatidylcholine, cholesterol, monolauroyl lysine, and propylene glycol) combined with a submicron emulsion phase comprising vegetable oil, surfactant, fatty alcohol, glycerol ester, and wax [233]. Foldvari et al. observed that the encapsulation of prostaglandin E1 in a biphasic vesicle system resulted in a significantly higher delivery through the skin and greater skin blood perfusion compared with traditional liposomal, non-liposomal and placebo formulations. The formulation increased the skin blood flow by tenfold compared with the non-liposomal PGE1 formulation and 25-fold compared with the liposomal placeb [234]. Further, lipid-based biphasic delivery system's efficacy has been assessed in delivering plasmid DNA to viable layers of human skin. Notably, three formulations exhibited significant dermal absorption: Formulation 26–3-2-DNA delivered approximately 1X10^9^ plasmid copies per cm^2^, while 17C3-1-DNA and 26–3-1-DNA yielded 5X10^6^ and 5X10^8^ copies per cm^2^, respectively [235]**.** The authors also developed a topical biphasic vesicle composition for IFNα and observed that there was a mean 12-fold increase of IFNα was seen in the skin after treatment, with the total amount of IFNα absorbed from the 3-cm diameter patch being about 60,000 units in the 40 MIU dose group. A reduction was also observed in the wart area index and HPV DNA burden in wart biopsies after applying the formulation [236]. King et al. developed Biphasix, a novel transdermal lipid-based system, to deliver macromolecules through the skin. They assessed its efficacy by administering insulin via transdermal patches to diabetic rats. The outcomes revealed a significant 43.7% decline in blood glucose levels, which lasted for 51.5 h, and a corresponding rise in serum insulin. Notably, the transdermal Biphasix-insulin patches exhibited a bioavailability of 21.5% (based on serum insulin) and 39.5% (based on blood glucose-lowering effects) [237]. In another study, Babiuk et al. developed biphasic vesicles containing a plasmid encoding bovine herpesvirus type-1 glycoprotein D (pgD). Their findings showed that this formulation led to a five-fold increase in anti-gD IgG levels compared to untreated animals or those administered naked DNA. Biphasix, a lipid-based delivery system, facilitated gene expression in lymph nodes when the plasmids were administered topically, while intradermal injection resulted in antigen expression in the skin. Gene gun delivery method, on the other hand, led to antigen expression in both the skin and draining lymph nodes. Through transcutaneous immunization with pgD in biphasic lipid vesicles, gD-specific antibody responses and a Th2-type cellular response were stimulated [238].

### Nanogels

Nanogels are nanoscale hydrogel particles forming a three-dimensional network structure, capable of delivering macromolecules like DNA, proteins, and drugs [239]. Made of crosslinked polymers, nanogels have unique attributes perfect for topical macromolecule delivery. They shield the cargo from degradation, improve stability, and boost therapeutic efficacy. Their small size efficiently penetrates the skin, enabling targeted delivery to specific skin layers [240]. The advantages of nanogels in topical macromolecule delivery encompass controlled release, sustained drug delivery, and minimal systemic exposure, thus reducing potential side effects [241]. Their biocompatibility and biodegradability ensure safe application. Additionally, nanogels can be engineered to respond to external stimuli, triggering release at specific sites and enhancing the solubility of hydrophobic drugs [242]. Nonetheless, some disadvantages exist. Complex synthesis may limit large-scale production and increase costs. Achieving uniform size distribution can be challenging, affecting stability and performance [243]. Kim et al. showed that by incorporating fluorescein isothiocyanate labelled bovine serum albumin into gelatin methacryloyl (GelMA) nanogels increases the permeation across the epidermis and into the dermis of porcine model in comparison to using fluorescein isothiocyanate labelled bovine serum albumin dissolved in phosphate buffer saline. Furthermore, the study investigated the transport processes, revealing that delivery of occurred through three penetration pathways (intercellular, follicular, and transcellular routes) [244]. In another study, Choi et al. demonstrated that chitosan-conjugated, Pluronic-based nanogels effectively transported hydrophilic proteins through human skin. In vitro permeation tests on human cadaver skin demonstrated increased permeability of different-sized hydrophilic proteins, such as FITC-BSA (67 kDa) and FITC-Insulin (6 kDa) via direct penetration. The chitosan-conjugated nanogel showed about three times higher penetration than the unmodified form after 24 h. When proteins were introduced alone, with chitosan, or in chitosan-conjugated Pluronic micelles, minimal permeation occurred [66]. In another study, Witting et al. developed protein-loaded thermoresponsive poly(N-isopropylacrylamide)-polyglycerol-based nanogels with a thermal trigger point at 35 °C. The skin penetration studies demonstrated that there was an efficient intraepidermal protein delivery, particularly in barrier-deficient skin, which was further proved by preparing transglutaminase 1-loaded nanogels which were able to deliver the protein into transglutaminase 1-deficient skin models, resulting in the restoration of skin barrier function [245].

## Conclusion and perspective

The dermal and transdermal routes for macromolecule delivery present both obstacles and promising prospects.  Due to their high potency and specificity, peptides, proteins, and nucleic acids hold immense potential for treating a broad spectrum of diseases and could revolutionize medical treatments, particularly for chronic conditions that require long term medication regimens. However, their large size and hydrophilicity present significant hurdles in crossing the skin barrier, primarily the stratum corneum. The development of adjuvants, peptides, mechanical enhancements, and nano-systems is a testament to the innovative approaches scientists are employing to overcome these challenges. Chemical penetration enhancers and hyaluronic acid-based adjuvants are particularly promising, as they modify the skin barrier properties to facilitate drug permeation. Peptides, on the other hand, offer advantage by aiding in delivery and enhancing penetration. Mechanical methods such as iontophoresis, ultrasound, and microneedles provide alternative strategies by physically breaching the skin barrier. Iontophoresis, which uses electric currents, and ultrasound, which uses sound waves, can significantly enhance the delivery of macromolecules. Microneedles create microchannels in the skin, allowing for more efficient drug transport without causing significant pain or discomfort. While these methods offer significant potential, their clinical translation is often hindered by the requirement for specialized equipment and the risk of skin irritation. On the other hand, nanotechnology, with its ability to create nanocarriers like liposomes and solid lipid nanoparticles, offers another promising avenue. These carriers protect macromolecules from enzymatic degradation and provide controlled release, thereby enhancing their stability and therapeutic efficacy. They also offer precise targeting and reduce off-target effects. Future research should not only focus on optimizing these delivery methods to enhance safety and efficacy but also address critical challenges such as scalability for mass production and regulatory hurdles that often delay clinical translation. The integration of these technologies, along with advancements in smart materials and formulation design, could overcome current limitations and enable the development of personalized and targeted therapies. Additionally, understanding long-term safety implications and patient compliance will be crucial for real-world adoption. Exploring novel strategies, such as combining different techniques and creating hybrid delivery platforms, may unlock new opportunities for improving therapeutic outcomes. In conclusion, while significant challenges remain in the dermal and transdermal delivery of macromolecules, the potential benefits make this a highly worthwhile pursuit. Ongoing research and innovation in transdermal delivery hold the potential to revolutionize treatment approaches, enhancing patient care and therapeutic outcomes.

## Data Availability

The data supporting the findings of this study are available from the corresponding author upon reasonable request.
